# Expression and prognostic roles of PRDXs gene family in hepatocellular carcinoma

**DOI:** 10.1186/s12967-021-02792-8

**Published:** 2021-03-26

**Authors:** Mingxing Xu, Jianliang Xu, Dun Zhu, Rishun Su, Baoding Zhuang, Ruiyun Xu, Lingli Li, Shuxian Chen, Yunbiao Ling

**Affiliations:** 1 Department of Hepatobiliary Surgery, The Third Affiliated Hospital of Sun Yat-Sen University, No. 600 Tianhe Road, Guangzhou, 510630 Guangdong China; 2 Department of Ultrasound Medicine, Banan District People’s Hospital of Chongqing, No. 2 Xinong Street, Yudong, Banan District, Chongqing, 401320 China; 3 Department of Surgery, Chaya People’s Hospital, Changdu, 854300 Tibet China

**Keywords:** PRDX family, HCC, Prognosis, Interaction network, Bioinformatics analysis

## Abstract

**Background:**

As the fourth leading cause of cancer-related death in the world, the therapeutic effect and 5-year overall survival of hepatocellular carcinoma (HCC) are not optimistic. Previous researches indicated that the disorder of PRDXs was related to the occurrence and development of cancers.

**Methods:**

In this study, PRDXs were found in various tumor cell lines by CCLE database analysis. The analysis results of UALCAN, HCCDB and Human Protein Atlas databases showed the expression of PRDXs mRNA and protein in HCC tissues was dysregulated. Besides, UALCAN was used to assess the correlations between PRDXs mRNA as well as methylation levels and clinical characterization.

**Results:**

High expression of PRDX1 or low expression of PRDX2/3 suggested poor prognosis for HCC patients which was demonstrated by Kaplan–Meier Plotter. The genetic alterations and biological interaction network of PRDXs in HCC samples were obtained from c-Bioportal. In addition, LinkedOmics was employed to analyze PRDXs related differentially expressed genes, and on this basis, enrichment of KEGG pathway and miRNAs targets of PRDXs were conducted. The results indicated that these genes were involved in several canonical pathways and certain amino acid metabolism, some of which may effect on the progression of HCC.

**Conclusions:**

In conclusion, the disordered expression of some PRDX family members was associated with the prognosis of HCC patients, suggesting that these PRDX family members may become new molecular targets for the treatment and prognosis prediction of HCC.

**Supplementary Information:**

The online version contains supplementary material available at 10.1186/s12967-021-02792-8.

## Background

Hepatocellular carcinoma (HCC) and intrahepatic cholangiocarcinoma (ICC) are two main types of primary hepatocellular carcinoma (PHC), of which HCC accounts for about 80% [1]. HCC can be caused by chronic infection of hepatitis B virus (HBV) or hepatitis C virus (HCV), alcoholism, metabolic syndrome associated with diabetes or obesity, and non-alcoholic fatty liver disease (NAFLD) [2]. In recent years, the incidence and mortality of HCC have been increasing in North America and several European regions, while in traditional high-risk areas, including Japan and parts of China, the incidence and mortality of HCC have been declining [3]. With approximately 782,000 deaths each year, HCC remains the fourth leading cause of cancer-related deaths worldwide [4]. The survival of HCC patients depends on a variety of factors, including Eastern Cooperative Oncology Group performance status, the presence of other complications, liver dysfunction, tumor burden, macrovascular invasion and metastasis [5].

In developed countries, 40–50% of patients have been diagnosed with early HCC due to surveillance programs, and are in a stage of potentially curative treatment [2]. However, the possibility of metastasis is high even after potential radical treatment [6], and the overall 5-year survival rate after developing HCC is only 50–70% [7]. In addition, patients with advanced HCC at the time of diagnosis cannot undergo surgical resection, orthotopic liver transplantation, or local percutaneous tumor ablation [8], and are not sensitive to chemotherapy or radiotherapy [9]. Therefore, patients with advanced HCC have a lower overall survival rate. Although the treatment of HCC has improved over the past decade, HCC, as an aggressive malignant tumor, is still highly resistant and difficult to treat. Therefore, it is necessary to find novel molecular targets that can improve the treatment of HCC or predict the prognosis of HCC patients.

Peroxiredoxins (PRDXs) are now considered to be a major superfamily of peroxidases conserved during the evolution of bacteria, archaea and eukaryotes [10]. Six mammalian isoforms of PRDXs have been identified, all of which are 22–27 kDa small proteins [11]. PRDXs have constitutive expression in almost all tissues and cell types, although they differ in expression levels, and also exist in the extracellular environments, [10]. PRDXs can reduce peroxide through catalyzing the oxidation of cysteine to sulfenic acid [12]. According to the position of cysteine residues in the catalytic reactions, they can be divided into three subgroups: typical 2-Cys: PRDX1-4; atypical 2-Cys: PRDX5 and 1-Cys: PRDX6 [11]. Among them, PRDX1, expressed in the cytoplasm of cells, is identified as the stress-induced macrophage protein produced by mouse peritoneal macrophages exposed to oxidative stress [13]. Extracellular PRDX1 plays a critical role in inflammatory regulation. It can be used as a molecular chaperone to regulate the actions of multitudinous molecules, or act as a regulator of transcription [14]. PRDX2, the peroxidase activity of which is activated by CDK2, inhibits the differentiation of acute myeloid leukemia (AML) cells [15]. PRDX4 controls neurogenesis by coupling the GDE2 surface expression in response to the redox environment in the endoplasmic reticulum [16]. Some research have shown that although PRDXs do not suppress cell proliferation, the loss of PRDX1 in mice results in tumorigenesis [11].

Currently, there are few studies on the role of PRDXs in HCC and the prognosis of patients with HCC. In this study, we analyzed data from public databases via various online analysis tools to explore the expression and influence of PRDXs in HCC, especially its predictive role in the prognosis of HCC patients.

## Methods

### CCLE analysis

Cancer Cell Line Encyclopedia (CCLE) (https://portals.broadinstitute.org/ccle) provides public access to genomic data, analysis and visualization for 1457 cell lines. Each gene has multiple datasets and data identifiers. The five major dataset types are Copy Number, mRNA expression (Affy), RPPA, RRBS, and mRNA (RNAseq). In this study, the mRNA expression (RNAseq) data of PRDX family in a series of cancer cell lines were used.

### UALCAN analysis

UALCAN (http://ualcan.path.uab.edu/analysis.html) is a comprehensive, friendly and interactive web resource to provide easy access to data from the Cancer Genome Atlas (TCGA) project. TCGA contains clinical data of 31 cancer types. The website can not only query and analyze the relative expression of genes between cancer samples and normal samples, but also can be based on the relative expression of individual cancer stage, tumor grade or other clinical pathological characteristics in different tumor subgroups. In this study, HCC samples from the TCGA were used to analyze the expression of PRDXs in HCC and normal tissues. The correlations between the expression of PRDXs and clinical pathological characteristics, including age, cancer stage, gender, and tumor grade were also accessed. Additionally, the correlations between the methylation levels of PRDXs and clinical pathological characteristics such as sample types, cancer stage and tumor grade were also evaluated.

### HCCDB analysis

HCCDB is a web-based database, aiming at providing a one-stop resource for gene expression atlas in HCC. Fifteen public dataset sets of HCC gene expression were archived in the HCCDB database (http://lifeome.net/database/hccdb/home.html), including 3917 samples. The database can be used for the identification of consistently differentially expressed genes in HCC, i.e. the function *t*-test in R is employed to detect whether there is significant difference in gene expression between tumor samples and adjacent samples in each dataset, followed by Benjamini–Hochberg correction. Genes detected in at least 8 datasets and significantly differentially expressed in at least half of the datasets containing these genes are identified as uniformly differentially expressed. HCCDB database was used to confirm whether PRDXs were significantly differentially expressed in HCC.

### Human Protein Atlas analysis

Human Protein Atlas (http://www.proteinatlas.org/) is a public database that allows free access to explore human proteome. It includes but not limited to Tissue Atlas, Cell Atlas and Pathology Atlas. Tissue Atlas consists of deep sequencing of RNA (RNAseq) from 37 major different normal tissue types and immunohistochemistry on tissue microarrays containing by 44 normal human tissue types. Cell Atlas provides a high-resolution insight into the spatial distribution of intracellular proteins, including mRNA expression profiles of 64 different human-derived cell lines, and details the subcellular distribution patterns of proteins in these cells. Pathology Atlas contains data on the expression of 17 major human cancer types from nearly 8000 patients. In addition, the corresponding proteins are analyzed by immunohistochemistry (IHC) to form a total of more than 5 million IHC cancer tissue images. In this study, only the immunohistochemical staining results of PRDXs in normal and pathological HCC samples were utilized.

### Kaplan–Meier plotter analysis

The Kaplan–Meier plotter (http://kmplot.com/analysis/) is capable to assess the effect of 54,000 genes on survival in 21 cancer types. Gene expression data, relapse free and overall survival information are downloaded from GEO, EGA and TCGA. According to various quantile expressions of the proposed biomarkers, the patient samples are divided into two groups. Kaplan–Meier survival plot is used to compare the two groups. The hazard ratio of 95% confidence intervals and logrank *P* value are calculated. In this study, the correlation between the mRNA levels of PRDX family members and overall survival of 364 patients with HCC was studied by Kaplan–Meier plotter. The hazard ratio (HR) and logrank *P* were presented in the results. *P* < 0.05 means statistical significance.

### c-BioPortal analysis

The cBio Cancer Genomics Portal (https://www.cbioportal.org/) is a comprehensive open resource, which can be used for interactive exploration of multiple cancer genomics datasets. Currently, there are 260 cancer studies and stores DNA copy number data (hypothesis and discrete values of each gene, such as “deep deletion” or “amplification” and log_2_ level), expression data of mRNA and miRNA, non-synonymous mutations, protein and phosphoprotein level (RPPA) data, DNA methylation data and some clinical data, etc. We analyzed genetic alterations of PRDXs in 372 HCC samples from TCGA. The search parameters included mutation, CNVs and mRNA expression. The tab OncoPrint shows an overview of genetic changes for each sample in PRDXs. The tab Network visualizes the biological interaction network of PRDXs in the public access database, and carries out color coding and screening options according to the frequency of genomic alterations of each gene.

### LinkedOmics analysis

LinkedOmics (http://www.linkedomics.org/login.php) is a publicly available portal that includes multi-omics data from 32 TCGA cancer types and mass spectrometry-based proteomics data generated by the Clinical Proteomics Tumor Analysis Consortium (CPTAC) for TCGA breast, colorectal and ovarian tumors. The web application has 3 analytical modules: LinkFinder, LinkInterpreter and LinkCompare. LinkFinder module was used to investigate differentially expressed genes related to PRDXs in TCGA LIHC (HCC) cohort (n = 371). Pearson Correlation test was performed for statistical analysis, and the results were presented in volcano plot and heat map. The data in LinkFinder results were signed and ranked. Then differentially expressed genes related to PRDXs were analyzed and enriched based on Kyoto Encyclopedia of Genes and Genomes (KEGG), Gene Ontology and other functional classifications by using GSEA of LinkInterpreter module. The rank criterion was false discovery rate (FDR) < 0.05, and 500 simulations were carried out.

## Results

### The expression of PRDXs in hepatocellular carcinoma from different databases

**Fig. 1:**
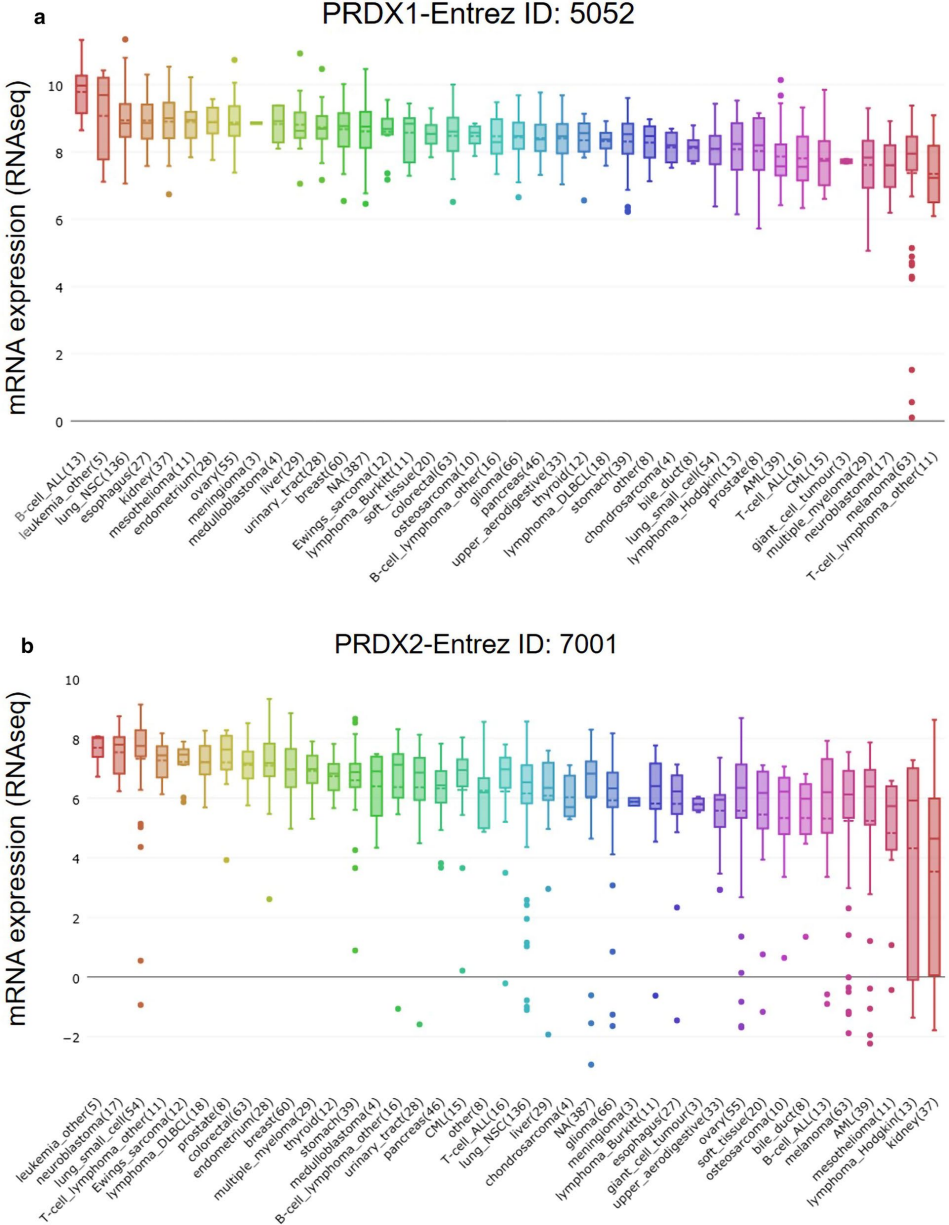
The mRNA expression levels of PRDX1 (**a**) and PRDX2 (**b**) in multiple common cancer cell lines were obtained from CCLE database. The dashed line within a box is the mean

**Fig. 2:**
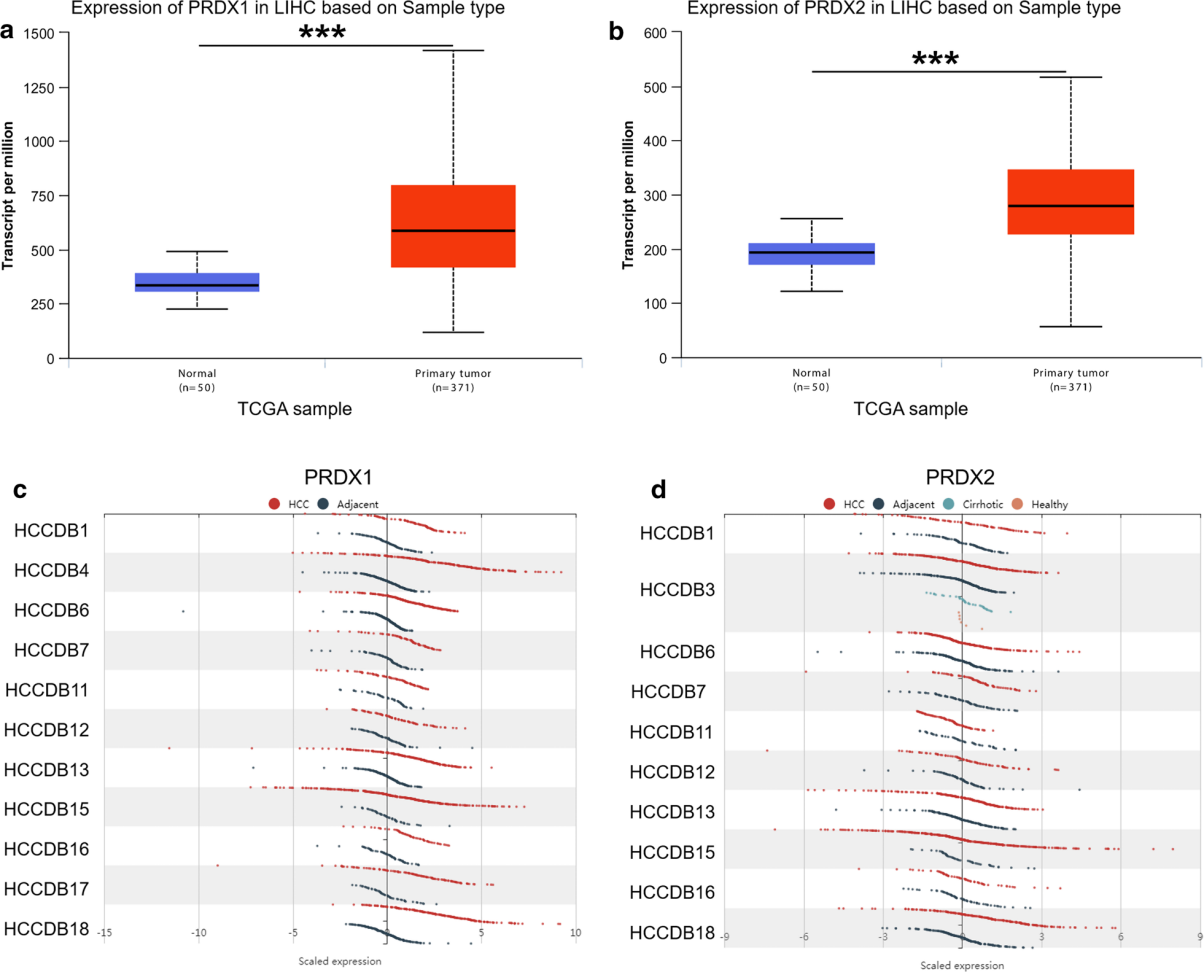
The relative expression of PRDXs in normal and HCC samples (UALCAN and HCCDB database). Boxplot showed the expression of PRDX1 (**a**) and PRDX2 (**b**) mRNA in HCC samples relative to normal samples based on TCGA database. The mRNA expression levels of PRDX1 (**c**) and PRDX2 (**d**) in different HCC datasets of HCCDB database were analyzed. LIHC: the abbreviation of liver hepatocellular carcinoma in TCGA database. Red: HCC samples; blue: adjacent normal tissue samples; cyan: cirrhotic samples; orange: healthy samples

The mRNA levels of PRDXs in various common cancer cell lines were obtained from CCLE database. The RNAseq results indicated that the mRNA levels of PRDXs in 29 liver cancer cell lines were maintained at 6–9, which was relatively higher compared with other cancer cell lines (Fig. 1a, b and Additional file 1: Figure S1A–D). Furthermore, the mRNA levels of PRDXs in HCC tissues were accessed by UALCAN database and compared with those in normal liver tissues. Among them, PRDX1 (Fig. 2a), PRDX2 (Fig. 2b), PRDX5 (Additional file 2: Figure S2C) and PRDX6 (Additional file 2: Figure S2D) were significantly upregulated in HCC while PRDX4 (Additional file 2: Figure S2B) was downregulated. There was no significant difference in the expression of PRDX3 between HCC and normal liver tissues (Additional file 2: Figure S2A).

Moreover, the mRNA expression of PRDXs in HCC, adjacent normal tissue, cirrhotic and healthy samples was obtained from HCCDB database. PRDX1 was confirmed to be upregulated in HCC tissues compared with adjacent normal tissues in all HCC datasets but HCCDB11 (Fig. 2c). PRDX2 was attested to have higher levels in HCC tissues in the HCCDB7 and HCCDB18 datasets (Fig. 2d). Nevertheless, both PRDX3 and PRDX4 were illustrated to be downregulated in HCC tissues by HCCDB1, HCCDB3, HCCDB4, HCCDB13, HCCDB15 and HCCDB16 dataset (Additional file 3: Figure S3A, B). PRDX5 was upregulated in HCC tissues in all datasets except for HCCDB11 and HCCDB16 (Additional file 3: Figure S3C). Results of HCCDB1, HCCDB4, HCCDB6, HCCDB15 and HCCDB17 datasets indicated downregulated of PRDX6 in HCC, while HCCDB7 dataset exhibited conversed results (Additional file 3: Figure S3D).

### Protein levels of PRDX gene family in HCC and the correlation between PRDXs expression and clinical characteristics

**Fig. 3:**
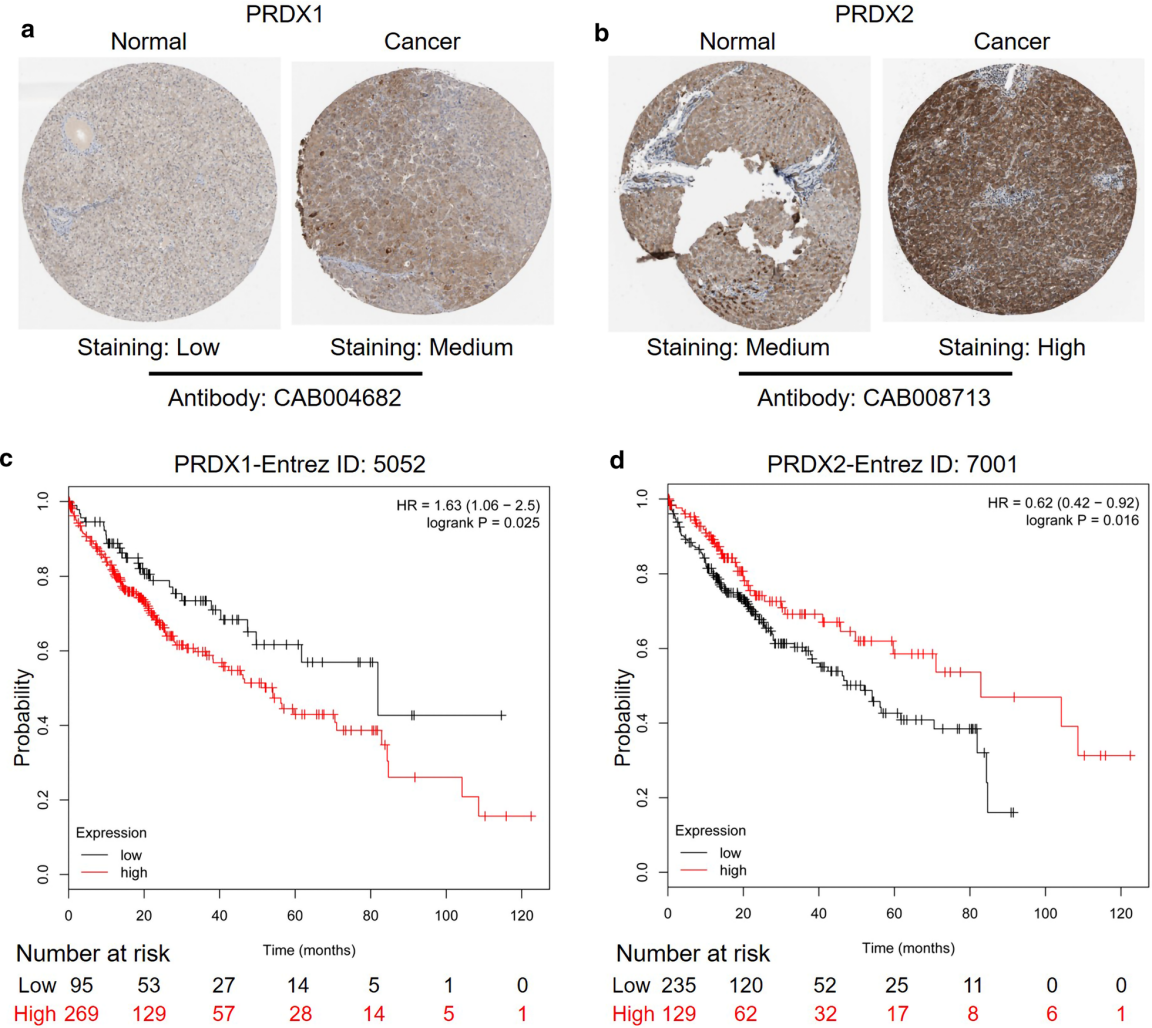
The protein levels of PRDXs and the correlations between PRDXs expression and patient prognosis in HCC. The protein levels of PRDX1 (**a**) and PRDX2 (**b**) in HCC tissues were compared with that in normal tissues by immunohistochemical staining. Kaplan–Meier Plotter database was utilized to evaluate the correlations between the expression of PRDXs (PRDX1 and PRDX2) and prognosis of HCC patients (**c**, **d**)

The protein expression of PRDXs was collected from the immunohistochemical staining results of the Human Protein Atlas database (Table 1), and several representative pictures of PRDXs in normal liver tissues and HCC tissues were selected and listed in Fig. 3a, b and Additional file 4: Figure S4A–D. In HCC tissues, the expression of PRDXs protein was mostly at a medium level, and occasionally at a low level. For example, 2 cases of PRDX4 and PRDX5 immunohichemical staining showed low level. Furthermore, based on the TCGA data from UALCAN database, the correlation analysis between PRDXs expression and age, cancer stage, gender, and tumor grade was further performed. Except for the dryregulated expression of PRDXs in HCC patients, the expression of some members of PRDX gene family was significant correlated with age (Additional file 11: Table S1), cancer stage (Additional file 12: Table S2), gender (Additional file 13: Table S3) and tumor grade (Additional file 14: Table S4). In addition, the correlation between PRDXs methylation levels in HCC and cancer stage as well as tumor grade was also analyzed. Among them, the methylation levels of PRDX1 and PRDX3 in HCC patients were obviously higher than those in normal people, whereas the methylation levels of PRDX2/4/5 were lower (Additional file 15: Table S5). Several significant correlations between PRDXs methylation levels and cancer stage (Additional file 16: Table S6) or tumor grade (Additional file 17: Table S7) were also observed.

**Table 1:** Immunohistochemical staining results of PRDXs protein obtained by Human Protein Atlas in normal liver tissues and hepatocellular carcinoma tissues

Genes	Normal	Cancer (cases)				Antibody
		High	Medium	Low	Not detected	
PRDX1	Low		6	3	1	CAB004682
PRDX2	Medium	5	3	4	1	CAB008713
PRDX3	High	5	7			CAB008656
PRDX4	Medium		9	2		CAB008659
PRDX5	Low		10	2		CAB008661
PRDX6	Medium		8	4		CAB008663

### Correlation between PRDXs levels and overall survival (OS) in patients with HCC

According to the analysis results of Kaplan–Meier Plotter, overexpression of PRDX1 was associated with poor prognosis in HCC patients (HR = 1.63) (Fig. 3c). However, low expression of PRDX2 (Fig. 3d) and PRDX3 (Additional file 5: Figure S5A) led to poor prognosis in HCC patients. There was no correlation between the expression of other PRDX family members (PRDX4/5/6) and the survival rate of HCC patients (Additional file 5: Figure S5B–D).

### Genomic alterations of PRDXs in HCC

**Fig. 4:**
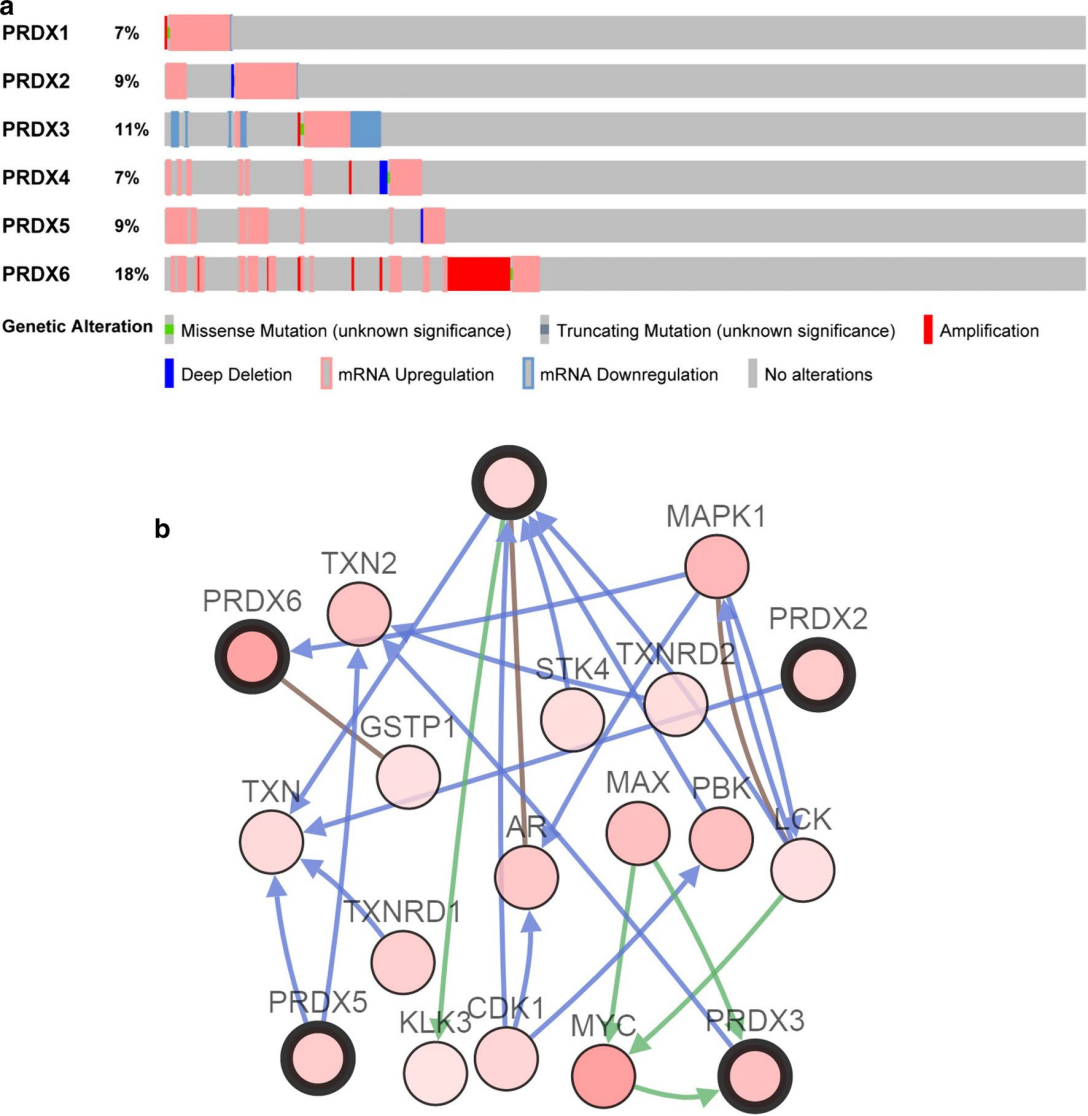
PRDXs alterations and biological interaction network in HCC. **a** The genetic alteration of PRDXs in HCC patients from TCGA were analyzed by c-BioPortal database. The different types of genetic alteration were highlighted in different color. **b** The network of PRDXs neighbor genes in HCC. PRDX1, PRDX2, PRDX3, PRDX5, and PRDX6 were the seed genes, indicated with thick border. The lighter red color suggested decrease frequency of genetic alteration in HCC

Based on sequencing data of HCC patients in TCGA database, the frequency and types of PRDXs alterations in HCC were monitored by cBioPortal (Fig. 4a). The results revealed that in 372 cases of HCC patients, PRDX1 and PRDX4 were altered in 26 cases (7%), PRDX2 and PRDX5 were altered in 33 cases (9%), PRDX3 was altered in 40 cases (11%), and PRDX6 was altered in 63 cases (18%). Moreover, the specific types and frequency of PRDXs alterations in HCC patients were shown in Table 1. Among these alterations, mRNA upregulation of PRDX1 was in 24 cases (6.4%), mRNA upregulation of PRDX2 was in 30 cases (8.1%), mRNA upregulation of PRDX3 was in 19 cases (5%), mRNA upregulation of PRDX5 was in 31 cases (8.3%), and mRNA upregulation of PRDX6 was in 33 cases (8.9%). Furthermore, the tab Network in cBioPortal was employed to reflect interactions between PRDXs and neighborhood genes in HCC (Fig. 4b). The neighbor genes of PRDXs with the most frequent alterations were MYC (18.6%), MAPK1 (13.1%) and PBK (11.4%) (Table 2).

**Table 2:** The type and frequency of PRDX gene family neighbor gene alterations in hepatocellular carcinoma (cBioPortal)

Genes	Amplification	Homozygous deletion	Up-regulation	Down-regulation	Mutation	Total alteration
AR			0.069	0	0.028	0.094
CDK1	0.006		0.064	0	0	0.069
EPX						
GPX1						
GSS						
GSTP1	0.025		0.025	0	0.006	0.053
KLK3	0.008		0.036	0	0	0.044
LCK	0.003	0.006	0.033	0	0.011	0.053
LPO						
MAPK1	0.003	0.003	0.047	0.069	0.008	0.131
MAX		0.003	0.053	0.058	0	0.111
MYC	0.178	0.003	0	0	0.006	0.186
PBK		0.058	0.053	0	0.003	0.114
PRDX1	0.003		0.064	0.003	0.003	0.072
PRDX2		0.003	0.081	0.003	0.006	0.092
PRDX3	0.003		0.05	0.053	0.006	0.111
PRDX4						
PRDX5		0.003	0.083	0	0	0.086
PRDX6	0.097		0.089	0	0.003	0.175
PXDN						
STK4	0.003		0.05	0	0.003	0.056
TXN	0.003		0.058	0	0.003	0.061
TXN2	0.008		0.083	0.008	0.008	0.106
TXNDC12						
TXNRD1	0.008		0.072	0	0	0.081
TXNRD2	0.006	0.003	0.047	0	0	0.056
TXNRD3						

### KEGG pathway analysis of PRDXs related differentially expressed genes correlated in HCC

**Fig. 5:**
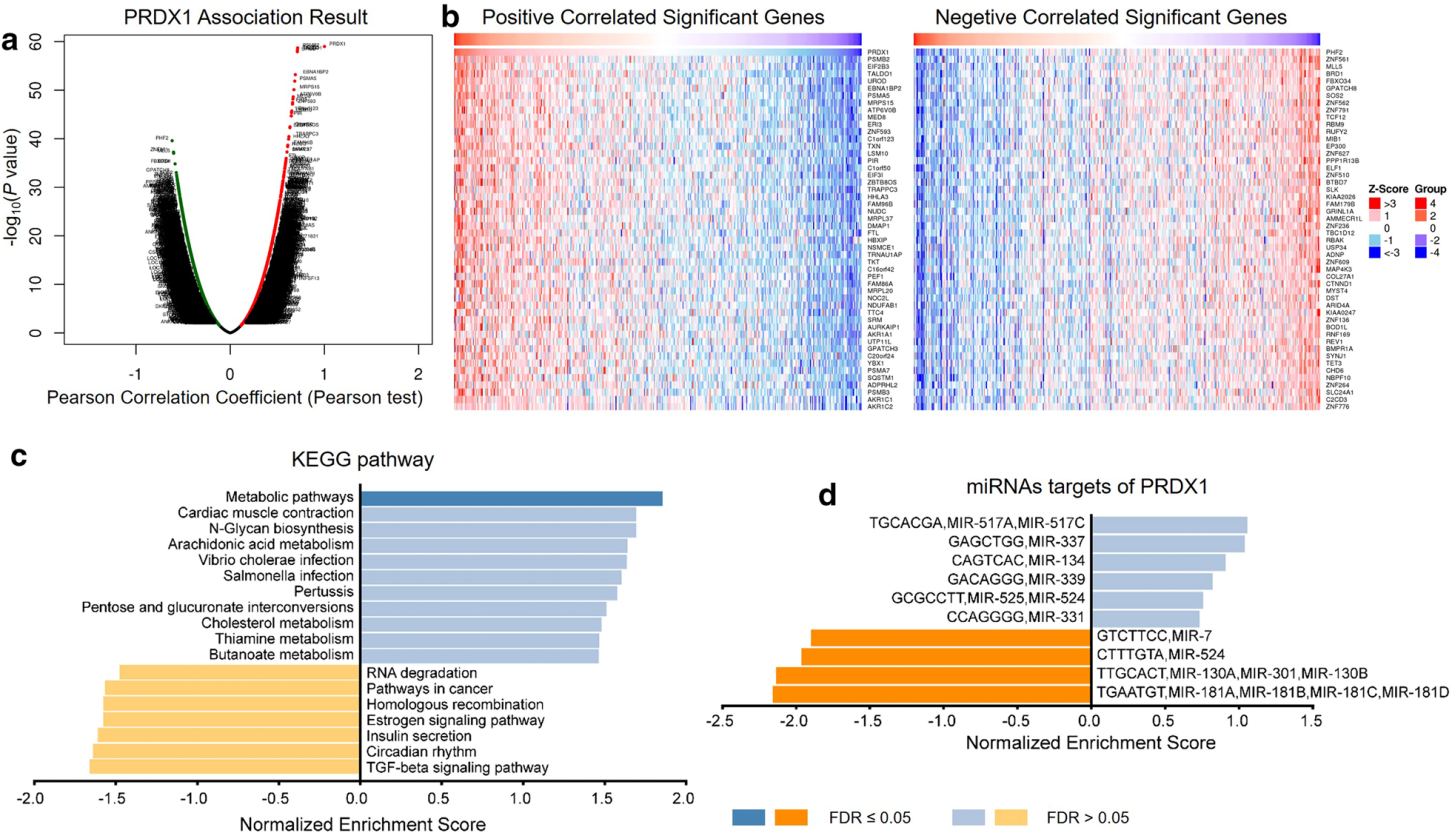
KEGG pathway enrichment analysis of PRDX1 co-expression genes and miRNA targets of PRDX1 in HCC. **a** Volcano plot showed the differential expression of genes related to PRDX1 in HCC and a Pearson correlation was used for the correlation analysis. Green: negatively correlated significant genes; red: positively correlated significant genes. **b** Top 50 positively and top 50 negatively correlated significant genes of PRDX1 were presented in the heat map. **c** The KEGG pathway enrichment of PRDX1 co-expression genes in HCC was analyzed using Gene Set Enrichment Analysis (GSEA). **d** The miRNA targets of PRDX1 in HCC. *FDR* false discovery rate

The LinkFinder module of LinkedOmics was used to analyze the mRNA sequencing data of 372 HCC patients in the TCGA. As shown by the volcano plot in Fig. 5a, 11,082 genes were negatively correlated with PRDX1 and 8840 genes were positively correlated with PRDX1. The top 50 positive significant genes and the top 50 negative significant genes correlated with PRDX1 were presented in the heat map in Fig. 5b. There was a strong negative correlation between PHF2 and PRDX1 (Pearson correlation = 0.62, *P* = 2.547e−40), whereas PSMB2 was strongly positively correlated with PRDX1 (Pearson correlation = 0.72, *P* = 1.961e−59). In addition, the specific analysis results of genes negatively or positively correlated with other PRDX family members were shown in panels A, B of Additional file 6: Figure S6, Additional file 7: Figure S7, Additional file 8: Figure S8, Additional file 9: Figure S9, Additional file 10: Figure S10. Combined with the above results, it was indicated that PRDXs had extensive influence on the transcriptome. Furthermore, the results of KEGG pathway analysis by gene set enrichment analysis (GSEA) revealed that differentially expression genes related to PRDX1 were mainly enriched in Metabolic pathways, TGF-beta signaling pathway, and so on (Fig. 5c). Besides, the enrichment results of differentially expressed genes in correlation with PRDX2, PRDX3, PRDX4, PRDX5 and PRDX6 showed that they were also involved in Metabolic pathways (panels C of Additional file 6: Figure S6, Additional file 7: Figure S7, Additional file 8: Figure S8, Additional file 9: Figure S9, Additional file 10: Figure S10). To further investigate the possible molecular mechanism of PRDX1 in HCC, the enrichment analysis of miRNAs by LinkInterpreter of LinkedOmics were utilized, and the results revealed that PRDX1 was associated with (GTCTTCC) MIR-7, (CTTTGTA) MIR-524, (TTGCACT) MIR-130A, MIR-301, MIR-130B (Fig. 5d). Furthermore, miRNA targets networks of the other members of PRDX family were also analyzed and showed in panel D of Additional file 6: Figure S6, Additional file 7: Figure S7, Additional file 8: Figure S8, Additional file 9: Figure S9, Additional file 10: Figure S10.

## Discussion

Hepatocellular carcinoma (HCC) is a relatively common cancer with increasing morbidity and mortality. About half of the new cases and deaths of HCC occurring annually in China [17]. Despite various treatments, the prognosis of HCC patients is poor, with a 5-year overall survival rate of less than 50%. However, through surgical excision or transplantation, the survival rate of HCC patients in early stage can reach 70% [18]. The only chance for long-term survival is early detection of tumors. Unfortunately, most HCC patients are diagnosed as advanced and ineligible for treatment [19].

It has been reported that the disorder of PRDX family was related to the occurrence and development of cancer [20]. To explore the role of PRDXs in the prognosis of HCC patients, the data from several public databases were analyzed and evaluated in this study through a variety of online tools. Firstly, according to the data from CCLE, PRDXs were expressed in multiple cancer cell lines, and the levels of PRDXs in HCC cell lines were mainly between 6 and 9. Knoops et al*.* also found that PRDXs were expressed in almost all tissues and cell lines [10]. In addition, the analysis of UALCAN database indicated that the mRNA expression of PRDX1/2/5/6 in HCC was significantly upregulated, while the mRNA expression of PRDX4 was downregulated. Similar results were obtained from the analysis of different HCC datasets in HCCDB. Immumohistochemical staining results in Human Protein Atlas database showed that PRDX1/2/5 were highly expressed in HCC tissues, while the expression of PRDX3/4 was low. This was similar to the results of Aguilar-Melero et al*.* research, that is, PRDX1 was highly expressed in various tumors including HCC [21]. Zhou et al*.* demonstrated that PRDX2 was often downregulated in HCC tissues, and played a tumorigenic role [22]. Xu et al. found that the expression level of PRDX6 in HCC tissues was lower than that in matched para-carcinoma tissues [23]. However, in these different databases, there were different analysis results about the expression of PRDX3 and PRDX6 in HCC, so we need to further study and verify in the follow-up experiments.

Kaplan–Meier survival analysis showed that high expression of PRDX1 and low expression of PRDX2/3 predicted poor prognosis in patients with HCC. The expression of other PRDXs in HCC was no significant correlation with prognosis. Previous studies have also confirmed that PRDX1 overexpression was associated with poor clinical prognosis of HCC [21], and Fang et al*.* revealed that decreased expression of PRDX1 in tumor tissues was an independent risk factor for overall survival and disease free survival in patients after surgery [24]. The downregulation of PRDX3 and PRDX6 was correlated with poor prognosis in HCC patients [23, 25]. Although the relationship between PRDX2 and prognosis of HCC patients has not been reported, the low expression of PRDX2 was confirmed to be associated with liver metastasis and poor overall survival rate of colorectal cancer [26]. It has been reported that high expression of PRDX4 was related to good tumor characteristics and prognosis of HCC patients [27], which was inconsistent with our findings and needed to be validated in experiments.

Furthermore, the types and frequency of PRDXs in HCC were determined based on the sequencing data of HCC patients in the cBioPortal. Among 372 cases of HCC patients, the patients with PRDX6 genetic alterations were the most, 63 cases (18%), followed by PRDX3, with 41 cases (11%); the patients with PRDX1 and PRDX4 genetic alterations were the least, 26 cases (7%) each. There were 24 cases with mRNA upregulation mutations of PRDX1, 19 cases with mRNA upregulation mutations of PRDX3, and 33 cases with mRNA upregulation mutations of PRDX6. More genetic alterations may be one reason why the expression levels of PRDX3 or PRDX6 are not consistent in different datasets. The tab network in cBioPortal was used to detect the adjacent genes of PRDXs, frequency of which were changed, with MYC (18.6%), MAPK1 (13.1%) and PBK (11.4%) having the most frequent changes in frequency. MYC is the main regulator of cell metabolism and proliferation, high constitutive expression of which drives many types of tumors, and often correlated with cancer invasion and poor prognosis [28]. Mitogen-activated protein kinase 1 (MAPK1), belonging to the MAP kinase family, is served as a binding site for many biochemical signals and involved in various cellular processes, such as cell differentiation, proliferation, transcriptional development and regulation [29]. PBK is a serine/threonine kinase belonging to the mitogen-activated protein kinase (MAPKK) family. PBK has a high trans-activity, plays a crucial role in various cancers, and also promotes migration and invasion of lung cancer cell [30]. These results suggested that PRDX1 may play a role in the prognosis of HCC by interacting with MYC, MAPK1 and PBK.

## Conclusions

LinkedOmics was employed to analyze the sequencing results of 372 HCC patients in TCGA database. We found that the pathways of PRDXs related gene enrichment mostly involved Metabolic pathway, as well as some classical pathways, such as NF-kappa B signaling pathway, TGF-beta signaling pathway and cell cycle. Studies from Zheng et al*.* revealed that PRDX1 knockdown significantly downregulated the level of β-catenin associated with WNT pathway, and then inhibited the occurrence of EMT [31]. Wang et al*.* proposed that PRDX2 served as a promoter of colon cancer stem cells properties via Hedgehog signaling pathway [32]. These results indicated that PRDXs had extensive effects on the transcriptome, and affected the progression of HCC through various downstream pathways. What’s more, PRDX2, as a target of miR-200b-3p, promoted the proliferation, invasion and EMT of colorectal cancer cells [33]; PRDX3, as a target molecule of miR-383, regulated the growth of medulloblastoma cells [34]. In this study, LinkInterpreter module of LinkedOmics was utilized to predict the interaction between PRDXs and miRNAs to preliminarily explore the regulatory mechanism of PRDXs in HCC. PRDX1 may target miR-517A/C, miR-339, miR-7 and other miRNAs.

In summary, several different online tools were used to analyze and evaluate HCC data from public databases in this study, which provided multilevel evidences for the expression of PRDX family members in HCC and their predictive role in the prognosis of HCC patients. The results showed that the dysregulation of PRDXs in HCC played a vital role in genome stability, prognosis, cell pathway and function. This study has the advantages of large sample size, low cost and easy operation. Nonetheless, the study still has some limitations of relatively single data sources: all the analyses in this study are based on the sequencing results of HCC patients in TCGA database. In addition, the analysis results of different online tools are not entirely consistent, and our results need to be further verified in experiments.

## Supplementary Information

**Additional file 1: Figure S1. **The mRNA expression levels of PRDX3 (**A**), PRDX4 (**B**), PRDX5 (**C**) and PRDX6 (**D**) in a variety of cancer cell lines were obtained from CCLE database. The dashed line within a box is the mean.

**Additional file 2: Figure S2. **Boxplot showed the relative expression of PRDXs in normal and HCC samples (UALCAN). Panels **A**–**D** represented for PRDX3, PRDX4, PRDX5 and PRDX6 mRNA expression in HCC samples relative to normal samples based on TCGA database.

**Additional file 3: Figure S3.** The mRNA expression levels of PRDX3 (**A**), PRDX4 (**B**), PRDX5 (**C**) and PRDX6 (**D**) in different HCC datasets of HCCDB database were analyzed. Red: HCC samples; blue: adjacent normal tissue samples; cyan: cirrhotic samples; orange: healthy samples.

**Additional file 4: Figure S4.** The protein levels of PRDXs in HCC tissues by Human Protein Atlas database. The protein levels of PRDXs (PRDX3-6) in HCC tissues were compared with that in normal tissues by immunohistochemical staining, shown in panels **A**–**D**.

**Additional file 5: Figure S5.** The correlations between PRDXs expression and patient prognosis in HCC. Kaplan–Meier Plotter database was utilized to evaluate the correlations between the expression of PRDXs (PRDX3-6) and prognosis of HCC patients (**A**–**D**).

**Additional file 6: Figure S6.** KEGG pathway enrichment analysis of PRDX2 co-expression genes and miRNA targets of PRDX2 in HCC. **A** Volcano plot showed the differential expression of genes related to PRDX2 in HCC and a Pearson correlation was used for the correlation analysis. Green: negatively correlated significant genes; red: positively correlated significant genes. **B** Top 50 positively and top 50 negatively correlated significant genes of PRDX2 were presented in the heat map. **C** The KEGG pathway enrichment of PRDX2 co-expression genes in HCC was analyzed using GSEA. **D** The miRNA targets of PRDX2 in HCC. FDR: false discovery rate.

**Additional file 7: Figure S7.** KEGG pathway enrichment analysis of PRDX3 co-expression genes and miRNA targets of PRDX3 in HCC. **A** Volcano plot showed the differential expression of genes related to PRDX3 in HCC and a Pearson correlation was used for the correlation analysis. Green: negatively correlated significant genes; red: positively correlated significant genes. **B** Top 50 positively and top 50 negatively correlated significant genes of PRDX3 were presented in the heat map. **C** The KEGG pathway enrichment of PRDX3 co-expression genes in HCC was analyzed using GSEA. **D** The miRNA targets of PRDX3 in HCC. FDR: false discovery rate.

**Additional file 8: Figure S8.** KEGG pathway enrichment analysis of PRDX4 co-expression genes and miRNA targets of PRDX4 in HCC. **A** Volcano plot showed the differential expression of genes related to PRDX4 in HCC and a Pearson correlation was used for the correlation analysis. Green: negatively correlated significant genes; red: positively correlated significant genes. **B** Top 50 positively and top 50 negatively correlated significant genes of PRDX4 were presented in the heat map. **C** The KEGG pathway enrichment of PRDX4 co-expression genes in HCC was analyzed using GSEA. **D** The miRNA targets of PRDX4 in HCC. FDR: false discovery rate.

**Additional file 9: Figure S9.** KEGG pathway enrichment analysis of PRDX5 co-expression genes and miRNA targets of PRDX5 in HCC. **A** Volcano plot showed the differential expression of genes related to PRDX5 in HCC and a Pearson correlation was used for the correlation analysis. Green: negatively correlated significant genes; red: positively correlated significant genes. **B** Top 50 positively and top 50 negatively correlated significant genes of PRDX5 were presented in the heat map. **C** The KEGG pathway enrichment of PRDX5 co-expression genes in HCC was analyzed using GSEA. **D** The miRNA targets of PRDX5 in HCC. FDR: false discovery rate.

**Additional file 10: Figure S10.** KEGG pathway enrichment analysis of PRDX6 co-expression genes and miRNA targets of PRDX6 in HCC. **A** Volcano plot showed the differential expression of genes related to PRDX6 in HCC and a Pearson correlation was used for the correlation analysis. Green: negatively correlated significant genes; red: positively correlated significant genes. **B** Top 50 positively and top 50 negatively correlated significant genes of PRDX6 were presented in the heat map. **C** The KEGG pathway enrichment of PRDX6 co-expression genes in HCC was analyzed using GSEA. **D** The miRNA targets of PRDX6 in HCC. FDR: false discovery rate.

**Additional file 11: Table S1.** The correlations of PRDXs mRNA expression with clinical indexes-patient age were analyzed by UALCAN database.

**Additional file 12: Table S2.** The correlations of PRDXs mRNA expression with clinical indexes-cancer stage were analyzed by UALCAN database.

**Additional file 13: Table S3.** The correlations of PRDXs mRNA expression with clinical indexes-gender were analyzed by UALCAN database.

**Additional file 14: Table S4.** The correlations of PRDXs mRNA expression with clinical indexes-tumor grade were analyzed by UALCAN database.

**Additional file 15: Table S5.** The correlations of PRDXs methylation level with sample type were analyzed by UALCAN database.

**Additional file 16: Table S6.** The correlations of PRDXs methylation with clinical indexes-cancer stage were analyzed by UALCAN database.

**Additional file 17: Table S7.** The correlations of PRDXs methylation level with clinical indexes-tumor grade were analyzed by UALCAN database.

## Data Availability

The datasets used and/or analysed during the current study are available from the corresponding author on reasonable request.
