# Supplementary figures and images for: Expression and prognostic roles of PRDXs gene family in hepatocellular carcinoma
Source: J Transl Med. 2021 Mar 26;19:126. doi: 10.1186/s12967-021-02792-8 (PMC7995729; doi:10.1186/s12967-021-02792-8)

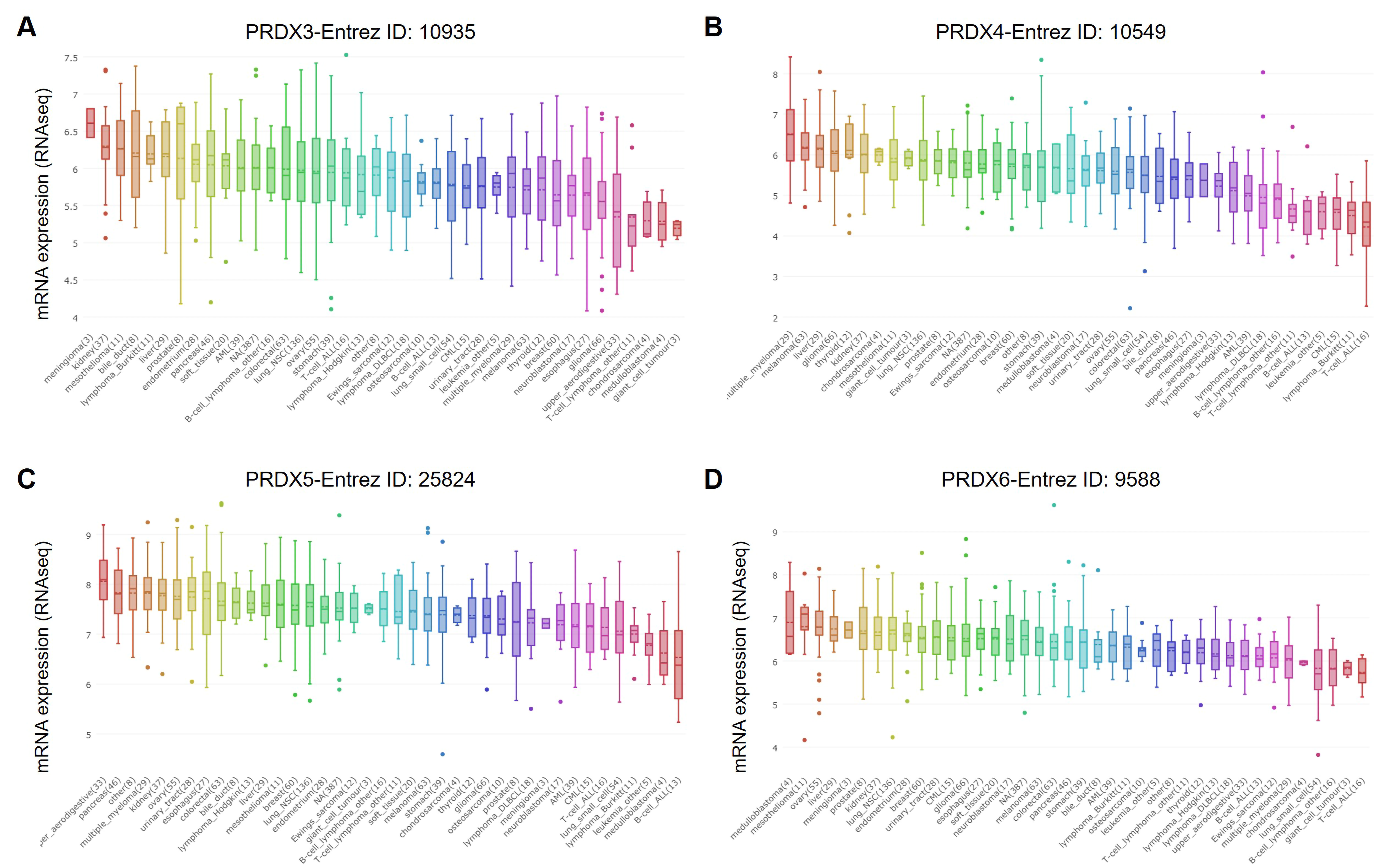

Supplement: Supplementary file 1 — Additional file 1: Figure S1. The mRNA expression levels of PRDX3 (A), PRDX4 (B), PRDX5 (C) and PRDX6 (D) in a variety of cancer cell lines were obtained from CCLE database. The dashed line within a box is the mean. [file 12967_2021_2792_MOESM1_ESM.tif]

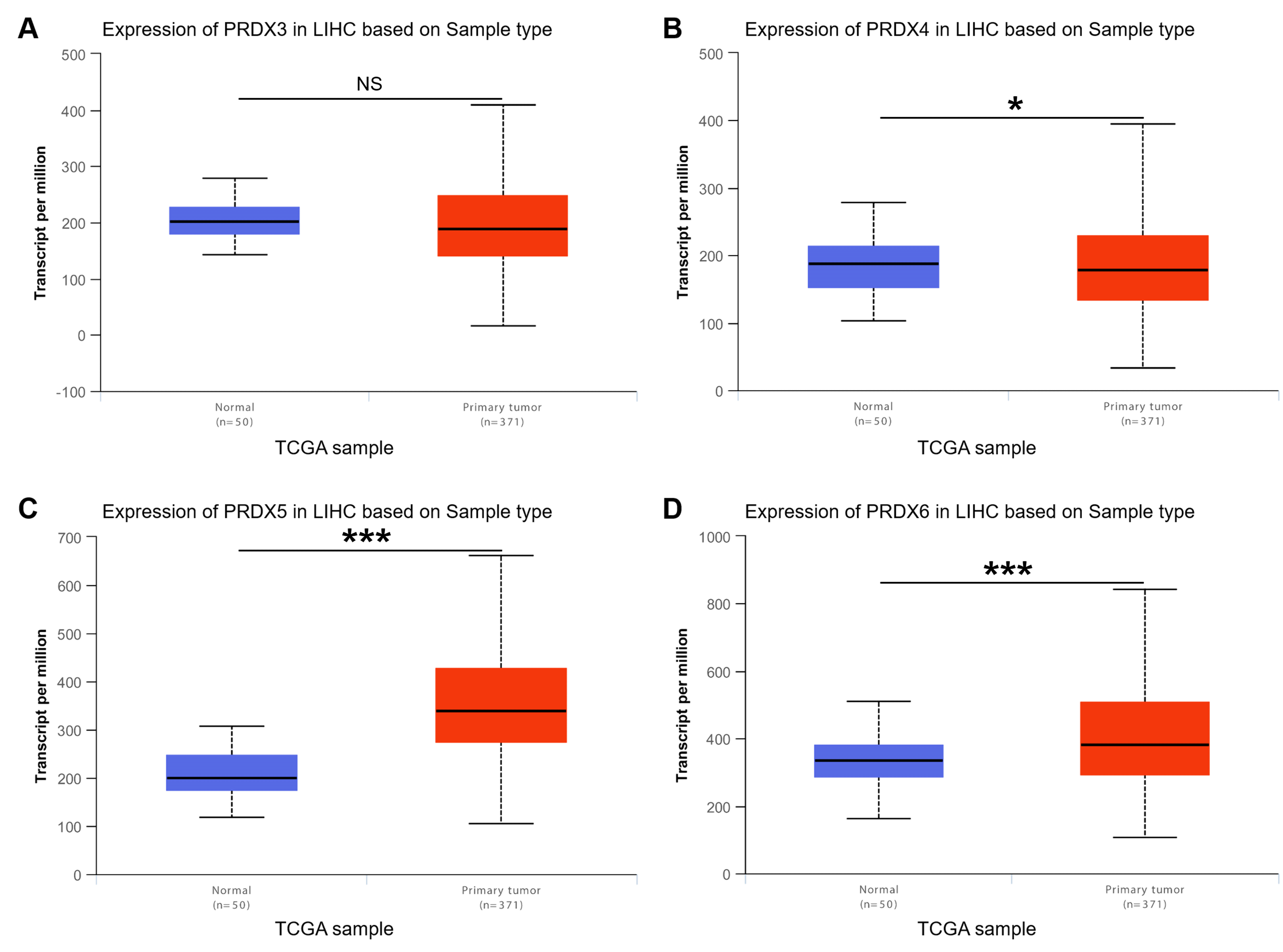

Supplement: Supplementary file 2 — Additional file 2: Figure S2. Boxplot showed the relative expression of PRDXs in normal and HCC samples (UALCAN). Panels A–D represented for PRDX3, PRDX4, PRDX5 and PRDX6 mRNA expression in HCC samples relative to normal samples based on TCGA database. [file 12967_2021_2792_MOESM2_ESM.tif]

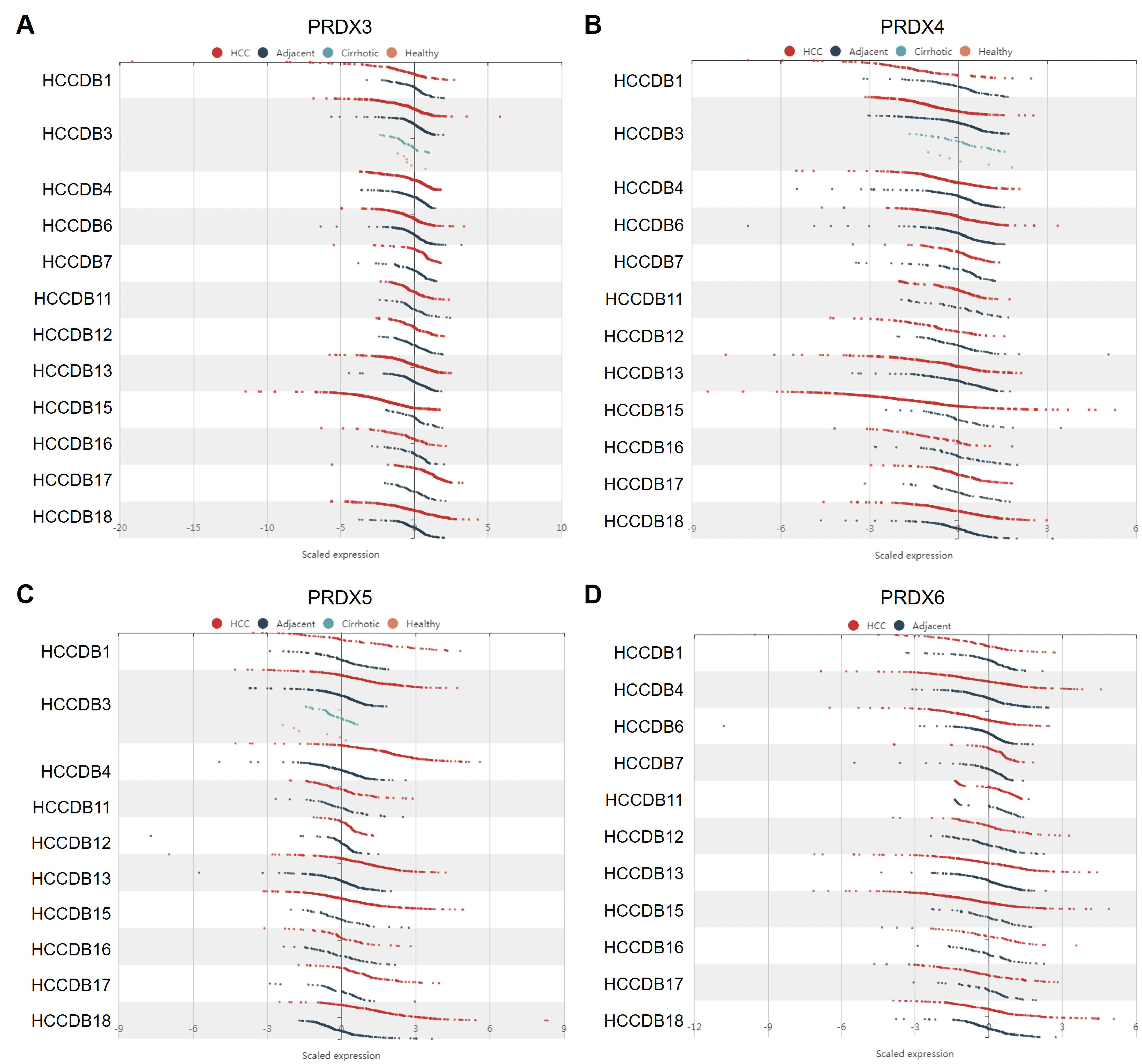

Supplement: Supplementary file 3 — Additional file 3: Figure S3. The mRNA expression levels of PRDX3 (A), PRDX4 (B), PRDX5 (C) and PRDX6 (D) in different HCC datasets of HCCDB database were analyzed. Red: HCC samples; blue: adjacent normal tissue samples; cyan: cirrhotic samples; orange: healthy samples. [file 12967_2021_2792_MOESM3_ESM.tif]

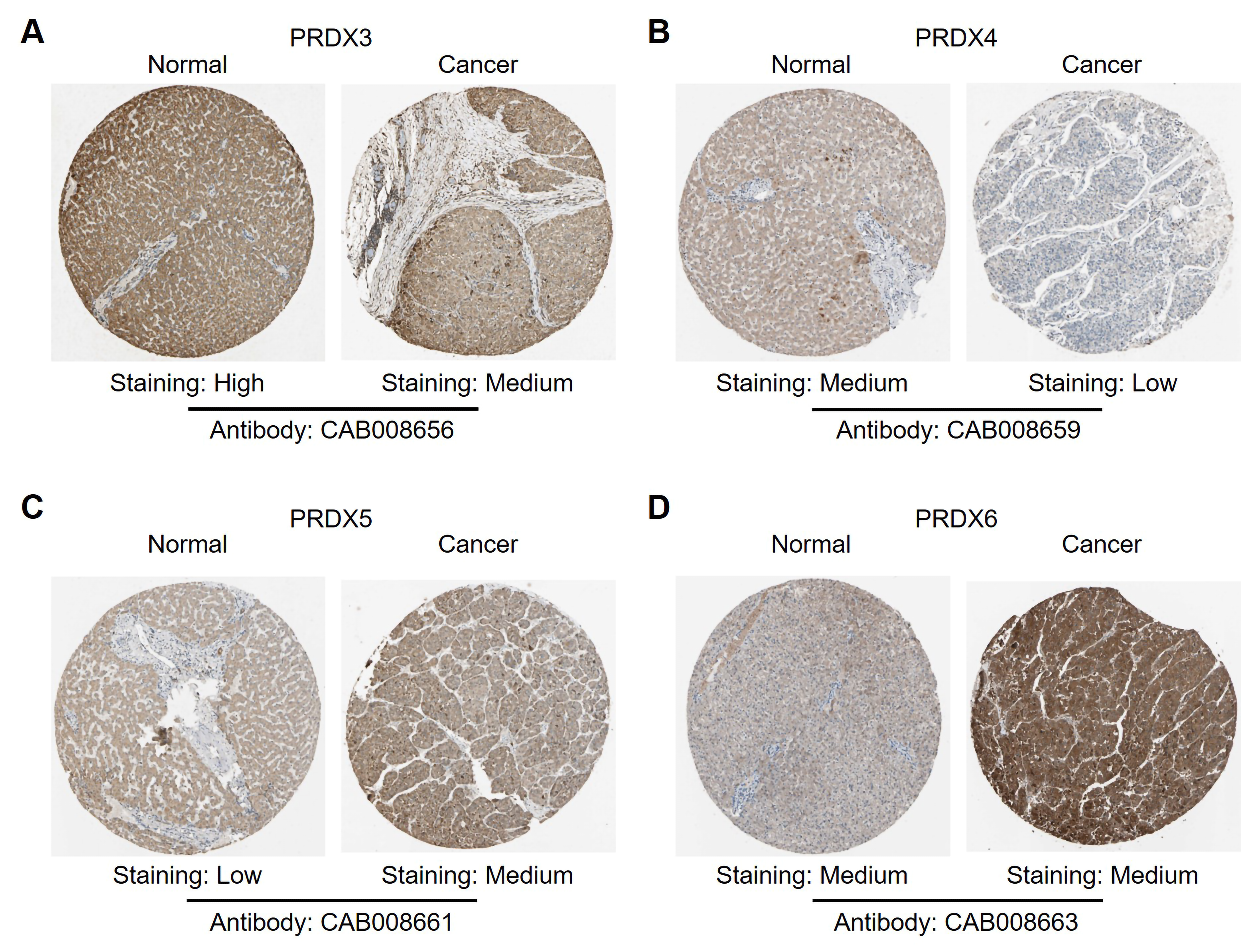

Supplement: Supplementary file 4 — Additional file 4: Figure S4. The protein levels of PRDXs in HCC tissues by Human Protein Atlas database. The protein levels of PRDXs (PRDX3-6) in HCC tissues were compared with that in normal tissues by immunohistochemical staining, shown in panels A–D. [file 12967_2021_2792_MOESM4_ESM.tif]

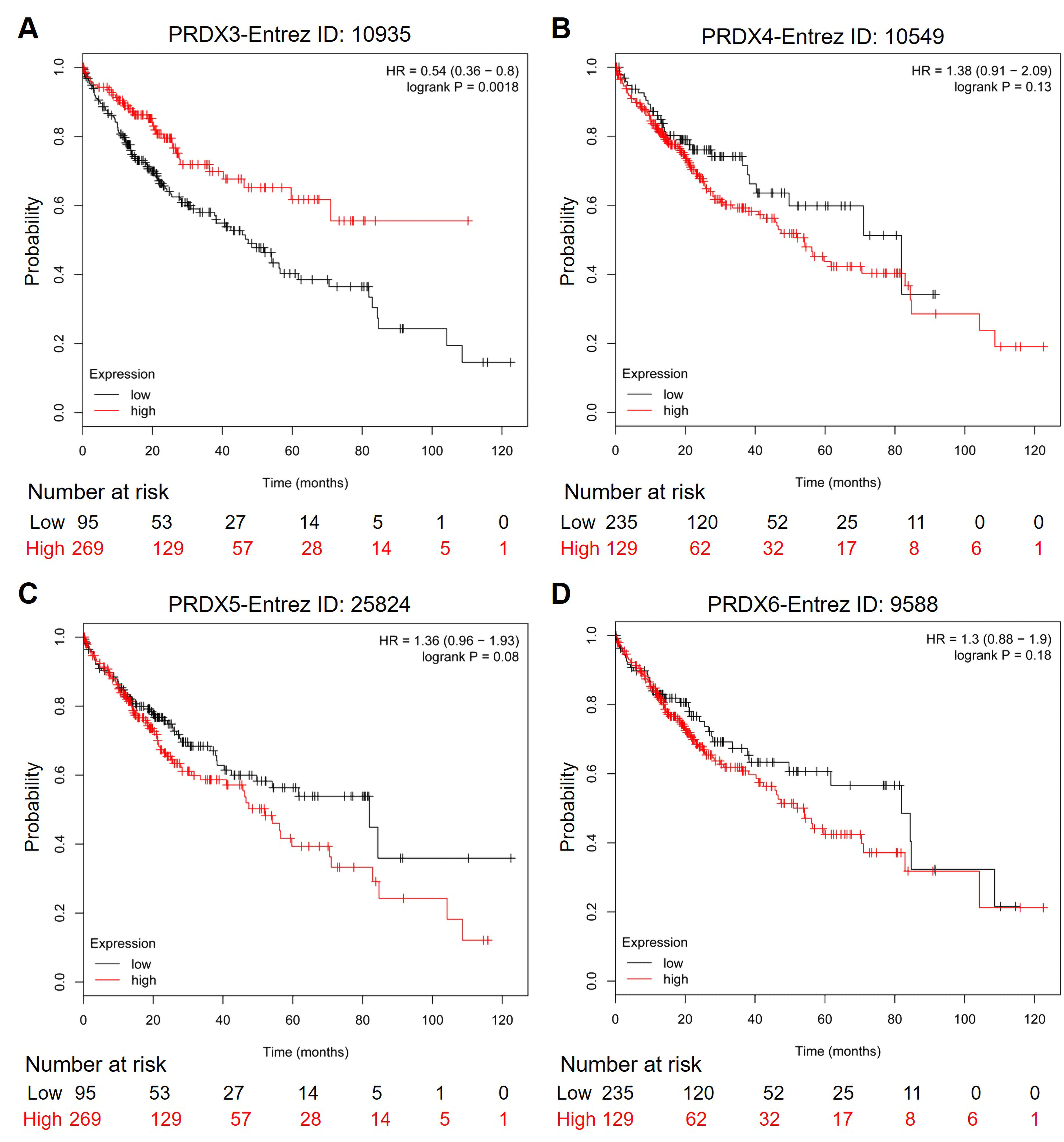

Supplement: Supplementary file 5 — Additional file 5: Figure S5. The correlations between PRDXs expression and patient prognosis in HCC. Kaplan–Meier Plotter database was utilized to evaluate the correlations between the expression of PRDXs (PRDX3-6) and prognosis of HCC patients (A–D). [file 12967_2021_2792_MOESM5_ESM.tif]

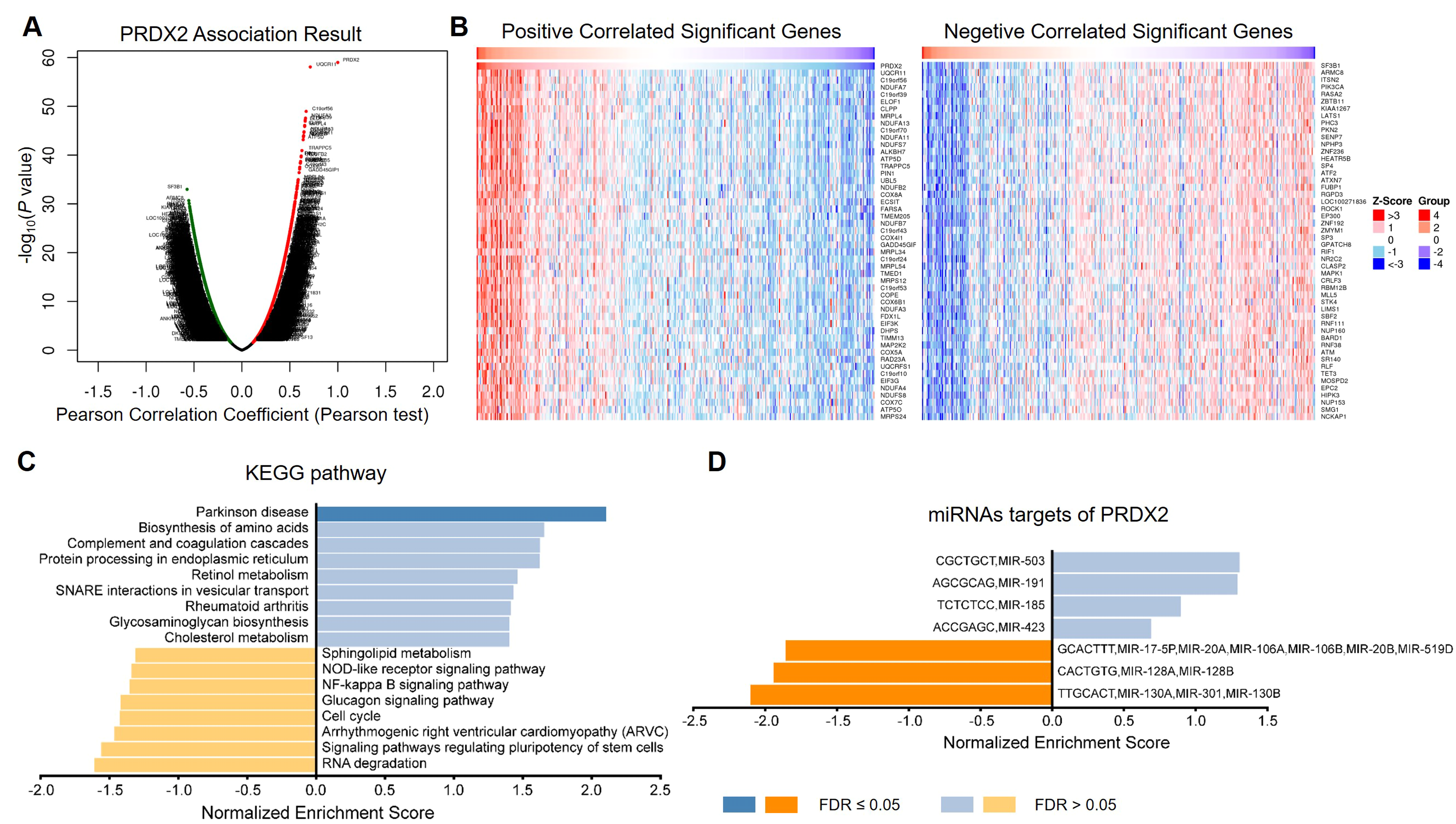

Supplement: Supplementary file 6 — Additional file 6: Figure S6. KEGG pathway enrichment analysis of PRDX2 co-expression genes and miRNA targets of PRDX2 in HCC. A Volcano plot showed the differential expression of genes related to PRDX2 in HCC and a Pearson correlation was used for the correlation analysis. Green: negatively correlated significant genes; red: positively correlated significant genes. B Top 50 positively and top 50 negatively correlated significant genes of PRDX2 were presented in the heat map. C The KEGG pathway enrichment of PRDX2 co-expression genes in HCC was analyzed using GSEA. D The miRNA targets of PRDX2 in HCC. FDR: false discovery rate. [file 12967_2021_2792_MOESM6_ESM.tif]

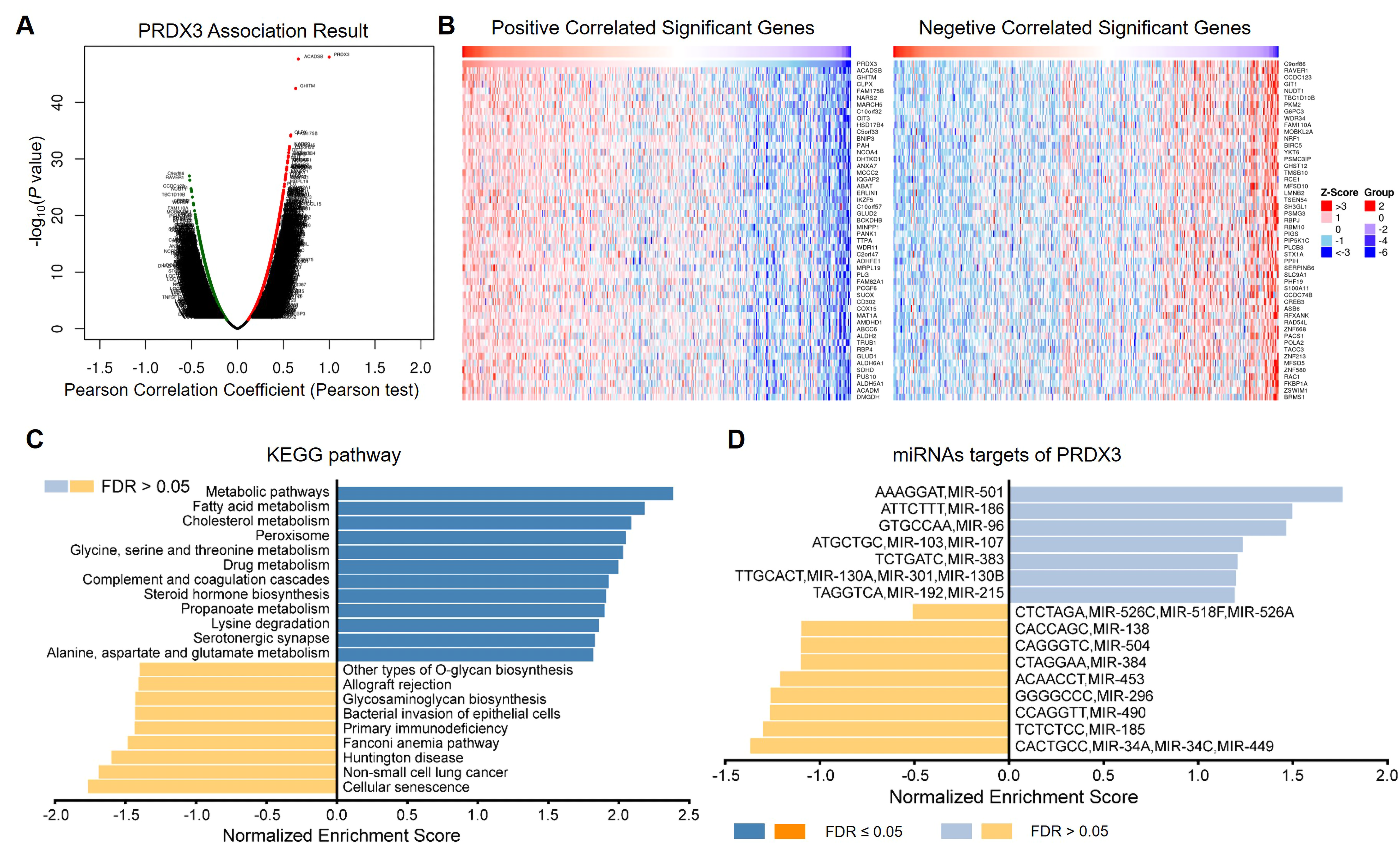

Supplement: Supplementary file 7 — Additional file 7: Figure S7. KEGG pathway enrichment analysis of PRDX3 co-expression genes and miRNA targets of PRDX3 in HCC. A Volcano plot showed the differential expression of genes related to PRDX3 in HCC and a Pearson correlation was used for the correlation analysis. Green: negatively correlated significant genes; red: positively correlated significant genes. B Top 50 positively and top 50 negatively correlated significant genes of PRDX3 were presented in the heat map. C The KEGG pathway enrichment of PRDX3 co-expression genes in HCC was analyzed using GSEA. D The miRNA targets of PRDX3 in HCC. FDR: false discovery rate. [file 12967_2021_2792_MOESM7_ESM.tif]

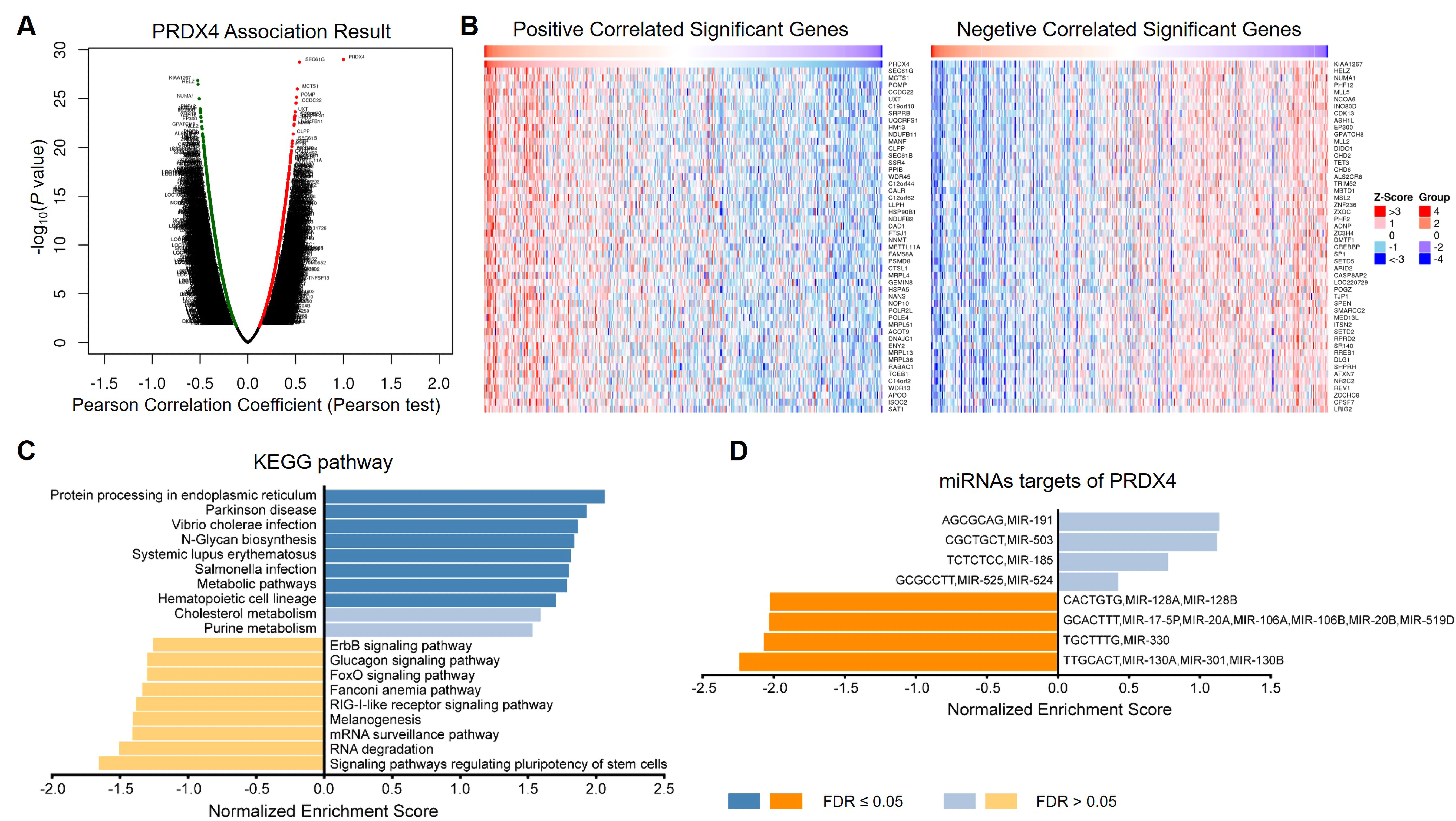

Supplement: Supplementary file 8 — Additional file 8: Figure S8. KEGG pathway enrichment analysis of PRDX4 co-expression genes and miRNA targets of PRDX4 in HCC. A Volcano plot showed the differential expression of genes related to PRDX4 in HCC and a Pearson correlation was used for the correlation analysis. Green: negatively correlated significant genes; red: positively correlated significant genes. B Top 50 positively and top 50 negatively correlated significant genes of PRDX4 were presented in the heat map. C The KEGG pathway enrichment of PRDX4 co-expression genes in HCC was analyzed using GSEA. D The miRNA targets of PRDX4 in HCC. FDR: false discovery rate. [file 12967_2021_2792_MOESM8_ESM.tif]

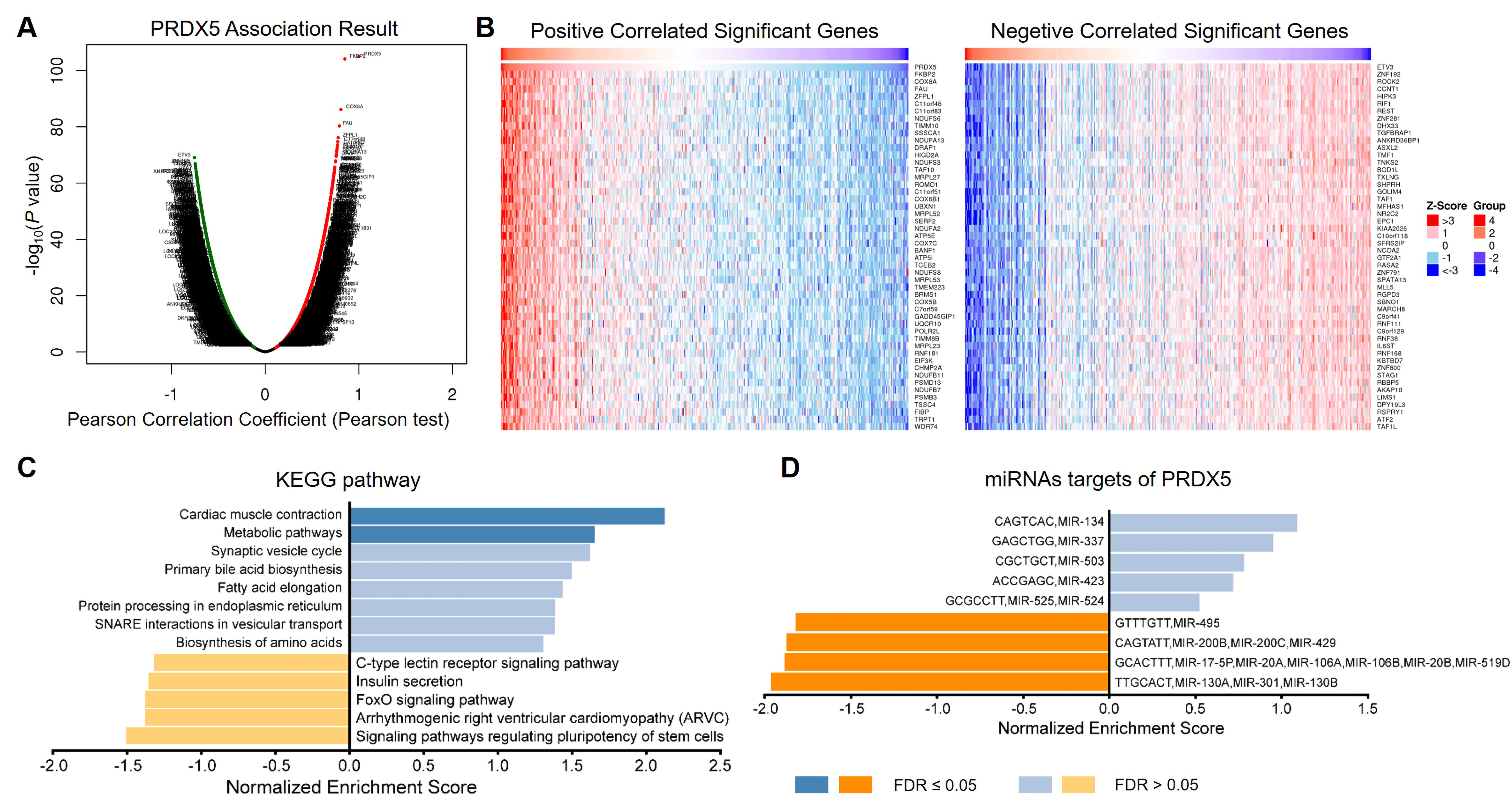

Supplement: Supplementary file 9 — Additional file 9: Figure S9. KEGG pathway enrichment analysis of PRDX5 co-expression genes and miRNA targets of PRDX5 in HCC. A Volcano plot showed the differential expression of genes related to PRDX5 in HCC and a Pearson correlation was used for the correlation analysis. Green: negatively correlated significant genes; red: positively correlated significant genes. B Top 50 positively and top 50 negatively correlated significant genes of PRDX5 were presented in the heat map. C The KEGG pathway enrichment of PRDX5 co-expression genes in HCC was analyzed using GSEA. D The miRNA targets of PRDX5 in HCC. FDR: false discovery rate. [file 12967_2021_2792_MOESM9_ESM.tif]

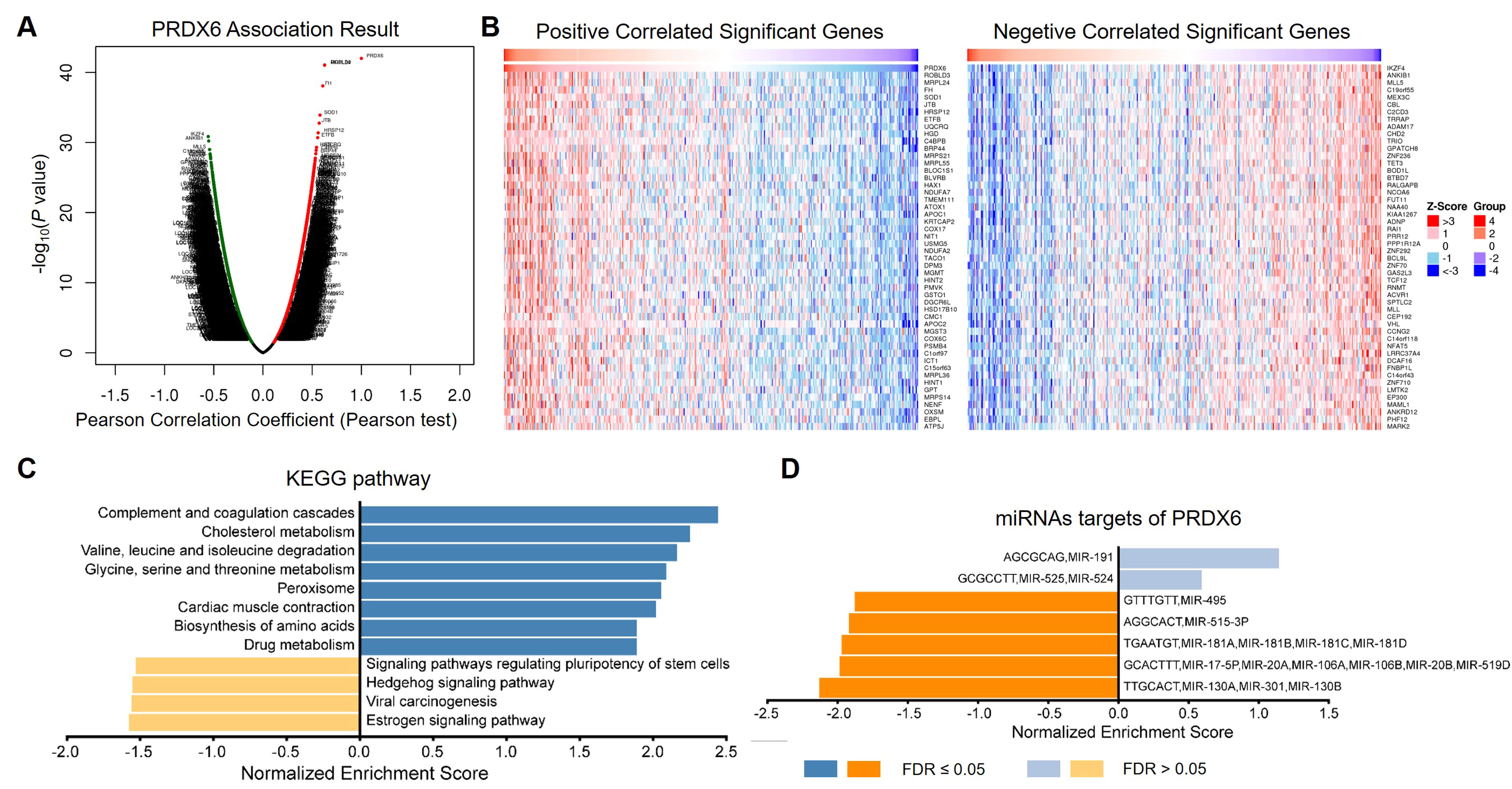

Supplement: Supplementary file 10 — Additional file 10: Figure S10. KEGG pathway enrichment analysis of PRDX6 co-expression genes and miRNA targets of PRDX6 in HCC. A Volcano plot showed the differential expression of genes related to PRDX6 in HCC and a Pearson correlation was used for the correlation analysis. Green: negatively correlated significant genes; red: positively correlated significant genes. B Top 50 positively and top 50 negatively correlated significant genes of PRDX6 were presented in the heat map. C The KEGG pathway enrichment of PRDX6 co-expression genes in HCC was analyzed using GSEA. D The miRNA targets of PRDX6 in HCC. FDR: false discovery rate. [file 12967_2021_2792_MOESM10_ESM.tif]
