# Supplementary material for: Expression and prognostic roles of PRDXs gene family in hepatocellular carcinoma
Source: J Transl Med. 2021 Mar 26;19:126. doi: 10.1186/s12967-021-02792-8 (PMC7995729; doi:10.1186/s12967-021-02792-8)
Supplement: Supplementary file 11 — Additional file 11: Table S1. The correlations of PRDXs mRNA expression with clinical indexes-patient age were analyzed by UALCAN database. [file 12967_2021_2792_MOESM11_ESM.docx]

**Table S1.** The correlations of PRDXs mRNA expression with clinical indexes-patient age were analyzed by UALCAN database.

| **Comparison** | **Statistical significance** | | | | | |
| --- | --- | --- | --- | --- | --- | --- |
|  | PRDX1 | PRDX2 | PRDX3 | PRDX4 | PRDX5 | PRDX6 |
| Normal vs Age  (21-40Yrs) | 1.64E-04 | 1.46E-06 | 3.02E-01 | 8.78E-02 | 4.90E-07 | 1.10E-02 |
| Normal vs Age  (41-60Yrs) | 4.66E-15 | 2.00E-14 | 1.89E-01 | 1.96E-01 | < 1E-12 | 1.62E-05 |
| Normal vs Age  (61-80Yrs) | < 1E-12 | < 1E-12 | 6.40E-01 | 1.10E-01 | 1.62E-12 | 1.01E-07 |
| Normal v Age  (81-100Yrs) | 2.50E-02 | 7.16E-03 | 3.42E-01 | 1.81E-01 | 2.70E-03 | 8.69E-01 |
| Age(21-40Yrs) vs  Age(41-60Yrs) | 8.14E-01 | 4.56E-01 | 8.51E-02 | 2.23E-01 | 1.95E-01 | 5.46E-01 |
| Age(21-40Yrs) vs  Age(61-80Yrs) | 9.45E-01 | 1.34E-01 | 1.84E-01 | 2.36E-01 | 2.09E-01 | 5.99E-01 |
| Age(21-40Yrs) vs  Age(81-100Yrs) | 5.60E-01 | 1.96E-02 | 1.93E-01 | 3.47E-02 | 1.12E-01 | 1.43E-01 |
| Age(41-60Yrs) vs  Age(61-80Yrs) | 7.18E-01 | 5.82E-01 | 3.86E-01 | 9.03E-01 | 8.50E-01 | 8.37E-01 |
| Age(41-60Yrs) vs  Age(81-100Yrs) | 4.97E-01 | 4.29E-02 | 7.08E-01 | 7.82E-02 | 3.96E-01 | 2.18E-01 |
| Age(61-80Yrs) vs  Age(81-100Yrs) | 4.70E-01 | 2.07E-01 | 4.89E-01 | 6.23E-02 | 3.39E-01 | 1.74E-01 |

Red indicates a statistically significant correlation.
