# Supplementary material for: Expression and prognostic roles of PRDXs gene family in hepatocellular carcinoma
Source: J Transl Med. 2021 Mar 26;19:126. doi: 10.1186/s12967-021-02792-8 (PMC7995729; doi:10.1186/s12967-021-02792-8)
Supplement: Supplementary file 12 — Additional file 12: Table S2. The correlations of PRDXs mRNA expression with clinical indexes-cancer stage were analyzed by UALCAN database. [file 12967_2021_2792_MOESM12_ESM.docx]

**Table S2.** The correlations of PRDXs mRNA expression with clinical indexes-cancer stage were analyzed by UALCAN database.

| **Comparison** | **Statistical significance** | | | | | |
| --- | --- | --- | --- | --- | --- | --- |
|  | PRDX1 | PRDX2 | PRDX3 | PRDX4 | PRDX5 | PRDX6 |
| Normal vs Stage1 | 1.63E-12 | 1.11E-16 | 8.05E-01 | 3.87E-01 | 1.62E-12 | 3.76E-07 |
| Normal vs Stage2 | 1.63E-12 | 5.41E-13 | 4.95E-01 | 1.15E-01 | 1.62E-12 | 1.00E-04 |
| Normal vs Stage3 | 1.57E-11 | 2.94E-13 | 2.31E-01 | 9.18E-02 | < 1E-12 | 6.64E-03 |
| Normal vs Stage4 | 1.43E-01 | 3.23E-02 | 8.60E-01 | 5.74E-02 | 4.64E-03 | 9.72E-07 |
| Stage1 vs Stage2 | 9.36E-02 | 7.09E-01 | 6.19E-01 | 2.94E-01 | 8.98E-02 | 4.70E-01 |
| Stage1 vs Stage3 | 4.22E-01 | 2.21E-01 | 3.35E-01 | 2.71E-01 | 6.37E-02 | 2.52E-01 |
| Stage1 vs Stage4 | 9.24E-01 | 9.57E-01 | 8.73E-01 | 4.78E-01 | 2.85E-01 | 3.63E-01 |
| Stage2 vs Stage3 | 4.87E-01 | 4.26E-01 | 7.06E-01 | 9.68E-01 | 8.12E-01 | 1.21E-01 |
| Stage2 vs Stage4 | 6.60E-01 | 9.45E-01 | 7.67E-01 | 6.90E-01 | 5.82E-01 | 6.06E-01 |
| Stage3 vs Stage4 | 8.78E-01 | 7.30E-01 | 6.35E-01 | 6.54E-01 | 7.04E-01 | 1.76E-01 |

Red indicates a statistically significant correlation.
