# Supplementary material for: Expression and prognostic roles of PRDXs gene family in hepatocellular carcinoma
Source: J Transl Med. 2021 Mar 26;19:126. doi: 10.1186/s12967-021-02792-8 (PMC7995729; doi:10.1186/s12967-021-02792-8)
Supplement: Supplementary file 13 — Additional file 13: Table S3. The correlations of PRDXs mRNA expression with clinical indexes-gender were analyzed by UALCAN database. [file 12967_2021_2792_MOESM13_ESM.docx]

**Table S3.** The correlations of PRDXs mRNA expression with clinical indexes-gender were analyzed by UALCAN database.

| **Comparison** | **Statistical significance** | | | | | |
| --- | --- | --- | --- | --- | --- | --- |
|  | PRDX1 | PRDX2 | PRDX3 | PRDX4 | PRDX5 | PRDX6 |
| Normal vs Male | 1.62E-12 | 1.62E-12 | 8.18E-01 | 7.14E-03 | 1.62E-12 | 8.55E-11 |
| Normal vs Female | 4.67E-11 | 1.63E-12 | 1.62E-01 | 7.39E-01 | 1.62E-12 | 3.36E-03 |
| Male vs Female | 3.99E-05 | 2.94E-02 | 2.28E-01 | 7.10E-03 | 2.07E-01 | 5.09E-02 |

Red indicates a statistically significant correlation.
