# Supplementary material for: Expression and prognostic roles of PRDXs gene family in hepatocellular carcinoma
Source: J Transl Med. 2021 Mar 26;19:126. doi: 10.1186/s12967-021-02792-8 (PMC7995729; doi:10.1186/s12967-021-02792-8)
Supplement: Supplementary file 14 — Additional file 14: Table S4. The correlations of PRDXs mRNA expression with clinical indexes-tumor grade were analyzed by UALCAN database. [file 12967_2021_2792_MOESM14_ESM.docx]

**Table S4.** The correlations of PRDXs mRNA expression with clinical indexes-tumor grade were analyzed by UALCAN database.

| **Comparison** | **Statistical significance** | | | | | |
| --- | --- | --- | --- | --- | --- | --- |
|  | PRDX1 | PRDX2 | PRDX3 | PRDX4 | PRDX5 | PRDX6 |
| Normal vs Grade1 | 2.45E-09 | 7.45E-11 | 9.83E-01 | 1.39E-01 | 2.02E-11 | 1.00E-04 |
| Normal vs Grade2 | 1.62E-12 | 1.62E-12 | 9.03E-01 | 2.22E-01 | < 1E-12 | 3.84E-04 |
| Normal vs Grade3 | 1.51E-14 | 1.63E-12 | 2.26E-01 | 1.16E-01 | < 1E-12 | 3.63E-08 |
| Normal vs Grade4 | 1.00E-03 | 3.29E-02 | 3.48E-01 | 3.37E-01 | 2.77E-04 | 2.75E-01 |
| Grade1 vs Grade2 | 2.20E-02 | 8.27E-01 | 9.19E-01 | 5.45E-01 | 5.38E-03 | 3.60E-02 |
| Grade1 vs Grade3 | 7.82E-05 | 3.37E-01 | 3.97E-01 | 9.41E-01 | 2.82E-03 | 2.63E-01 |
| Grade1 vs Grade4 | 2.88E-02 | 9.86E-01 | 2.81E-01 | 8.03E-01 | 1.09E-02 | 4.16E-01 |
| Grade2 vs Grade3 | 7.62E-03 | 3.97E-01 | 2.71E-01 | 5.50E-01 | 6.02E-01 | 7.80E-04 |
| Grade2 vs Grade4 | 3.53E-02 | 9.09E-01 | 3.63E-01 | 5.71E-01 | 3.40E-03 | 8.94E-01 |
| Grade3 vs Grade4 | 7.77E-01 | 6.87E-01 | 4.22E-01 | 8.06E-01 | 9.96E-03 | 2.72E-01 |

Red indicates a statistically significant correlation.
