# Supplementary material for: Expression and prognostic roles of PRDXs gene family in hepatocellular carcinoma
Source: J Transl Med. 2021 Mar 26;19:126. doi: 10.1186/s12967-021-02792-8 (PMC7995729; doi:10.1186/s12967-021-02792-8)
Supplement: Supplementary file 15 — Additional file 15: Table S5. The correlations of PRDXs methylation level with sample type were analyzed by UALCAN database. [file 12967_2021_2792_MOESM15_ESM.docx]

**Table S5.** The correlations of PRDXs methylation level with sample type were analyzed by UALCAN database.

| **Comparison** | **Statistical significance** | | | | | |
| --- | --- | --- | --- | --- | --- | --- |
|  | PRDX1 | PRDX2 | PRDX3 | PRDX4 | PRDX5 | PRDX6 |
| Normal vs Primary | 8.44E-10 | 3.78E-02 | 5.27E-13 | 1.03E-02 | 8.21E-05 | 8.88E-01 |

Red indicates a statistically significant correlation.
