# Supplementary material for: Expression and prognostic roles of PRDXs gene family in hepatocellular carcinoma
Source: J Transl Med. 2021 Mar 26;19:126. doi: 10.1186/s12967-021-02792-8 (PMC7995729; doi:10.1186/s12967-021-02792-8)
Supplement: Supplementary file 16 — Additional file 16: Table S6. The correlations of PRDXs methylation with clinical indexes-cancer stage were analyzed by UALCAN database. [file 12967_2021_2792_MOESM16_ESM.docx]

**Table S6.** The correlations of PRDXs methylation with clinical indexes-cancer stage were analyzed by UALCAN database.

| **Comparison** | **Statistical significance** | | | | | |
| --- | --- | --- | --- | --- | --- | --- |
|  | PRDX1 | PRDX2 | PRDX3 | PRDX4 | PRDX5 | PRDX6 |
| Normal vs Stage1 | 1.13E-10 | 2.78E-04 | 2.88E-10 | 2.42E-03 | 2.64E-04 | 8.76E-01 |
| Normal vs Stage2 | 2.62E-04 | 2.88E-03 | 9.66E-05 | 9.80E-03 | 2.56E-06 | 2.41E-01 |
| Normal vs Stage3 | 6.54E-03 | 8.89E-01 | 2.84E-03 | 4.96E-02 | 1.44E-04 | 6.99E-01 |
| Normal vs Stage4 | 1.83E-01 | 6.82E-03 | 8.72E-02 | 3.36E-01 | 5.55E-01 | 8.84E-01 |
| Stage1 vs Stage2 | 5.32E-01 | 9.37E-01 | 4.28E-01 | 4.68E-01 | 4.71E-02 | 2.32E-01 |
| Stage1 vs Stage3 | 8.36E-01 | 1.48E-01 | 1.35E-01 | 1.30E-01 | 7.59E-01 | 7.86E-01 |
| Stage1 vs Stage4 | 4.01E-01 | 6.84E-01 | 2.46E-01 | 7.19E-03 | 3.77E-01 | 8.10E-01 |
| Stage2 vs Stage3 | 5.99E-01 | 1.61E-01 | 5.33E-01 | 4.47E-01 | 9.49E-02 | 2.21E-01 |
| Stage2 vs Stage4 | 6.49E-01 | 6.94E-01 | 1.68E-01 | 2.30E-02 | 3.49E-02 | 9.21E-01 |
| Stage3 vs Stage4 | 2.75E-01 | 8.58E-02 | 1.31E-01 | 4.75E-02 | 1.76E-01 | 7.76E-01 |

Red indicates a statistically significant correlation.
