# Supplementary material for: Expression and prognostic roles of PRDXs gene family in hepatocellular carcinoma
Source: J Transl Med. 2021 Mar 26;19:126. doi: 10.1186/s12967-021-02792-8 (PMC7995729; doi:10.1186/s12967-021-02792-8)
Supplement: Supplementary file 17 — Additional file 17: Table S7. The correlations of PRDXs methylation level with clinical indexes-tumor grade were analyzed by UALCAN database. [file 12967_2021_2792_MOESM17_ESM.docx]

**Table S7.** The correlations of PRDXs methylation level with clinical indexes-tumor grade were analyzed by UALCAN database.

| **Comparison** | **Statistical significance** | | | | | |
| --- | --- | --- | --- | --- | --- | --- |
|  | PRDX1 | PRDX2 | PRDX3 | PRDX4 | PRDX5 | PRDX6 |
| Normal vs Grade1 | 3.21E-02 | 9.87E-11 | 2.74E-06 | 2.60E-02 | 3.78E-03 | 7.18E-02 |
| Normal vs Grade2 | 3.85E-07 | 1.42E-02 | 6.86E-08 | 5.52E-03 | 6.19E-05 | 3.46E-01 |
| Normal vs Grade3 | 4.62E-08 | 6.64E-02 | 2.14E-06 | 2.22E-02 | 1.59E-04 | 5.70E-03 |
| Normal vs Grade4 | 8.03E-05 | 1.11E-02 | 1.32E-01 | 8.76E-02 | 2.88E-03 | 2.21E-01 |
| Grade1 vs Grade2 | 5.07E-02 | 3.88E-01 | 2.45E-01 | 6.73E-01 | 1.55E-01 | 1.83E-01 |
| Grade1 vs Grade3 | 7.13E-01 | 4.67E-01 | 3.57E-01 | 7.88E-01 | 2.87E-01 | 1.52E-03 |
| Grade1 vs Grade4 | 7.48E-01 | 1.54E-01 | 4.31E-01 | 5.63E-01 | 4.96E-02 | 6.96E-02 |
| Grade2 vs Grade3 | 4.07E-01 | 6.61E-01 | 8.36E-01 | 3.66E-01 | 8.15E-01 | 7.18E-04 |
| Grade2 vs Grade4 | 5.48E-01 | 1.66E-01 | 8.67E-01 | 7.02E-01 | 1.54E-01 | 1.47E-01 |
| Grade3 vs Grade4 | 9.65E-01 | 2.55E-01 | 8.11E-01 | 5.09E-01 | 1.85E-01 | 7.38E-01 |

Red indicates a statistically significant correlation.
